# Plant Extracts Control In Vitro Growth of Disease-Causing Fungi in Chayote

**DOI:** 10.3390/plants12091800

**Published:** 2023-04-27

**Authors:** Edgar García-Ramírez, Adriana Contreras-Oliva, Josafhat Salinas-Ruiz, Gabriela Hernández-Ramírez, José Luis Spinoso-Castillo, Saira Itzel Colmenares Cuevas

**Affiliations:** 1 Postgraduate College Campus Cordoba, Federal Highway Cordoba-Veracruz, Km 348. Congregation Manuel Leon, Amatlan de los Reyes, Veracruz 94953, Mexico; 2 National Technological Institute of Mexico/Higher Technological Institute of Tierra Blanca, Av. Veracruz Esquina con Heroes de Puebla, Colonia Pemex, Tierra Blanca, Veracruz 95180, Mexico

**Keywords:** *Azadirachta indica*, *Cinnamomum zeylanicum*, *Diospyros digyna*, natural fungal control, in vitro evaluation, inhibition of mycelial growth

## Abstract

The use of agrochemicals has caused environmental problems and toxicity to humans, so natural alternatives for disease control during harvest and postharvest have been evaluated. The aim of this study was to evaluate cinnamon essential oil, neem oil, and black sapote fruit extract for in vitro inhibition of fungi isolated from chayote fruit. The extracts were applied at 300, 350, and 400 ppm in Petri dishes and the mycelial growth of *Fusarium oxysporum, Fusarium solani, Goetrichum* sp., and *Phytophthora capsici* was evaluated for 7 days, and the percentage of mycelial growth inhibition per day was calculated. Cinnamon oil showed a fungicidal effect at all concentrations. Neem oil at 400 ppm showed a 42.3% reduction in the growth of *F. solani* and 27.8% reduction in the growth of *F. oxysporum,* while at 350 ppm it inhibited the mycelial growth of *Phytophthora capsici* by 53.3% and of *Goetrichum* sp. by 20.9%; finally, the black sapote extract at 400 ppm inhibited 21.9–28.6% of the growth of all fungi. The growth of postharvest fungi on chayote fruit could be prevented or reduced by applying the plant extracts evaluated at adequate concentrations.

## 1. Introduction

Chayote fruit (*Sechium edule* (Jacq.) Swartz) is a species of the family Cucurbitaceae native to Mexico. The chayote fruit variety *virens levis*, known as smooth green chayote fruit, is a commercial crop whose fruit is mainly consumed as a vegetable. Mexico ranks first in the world as a producer and exporter of smooth green chayote fruit, followed by Costa Rica [1]. In 2019 alone, 195,191.52 t of chayote fruit was produced in Mexico, 85% of which was produced in the state of Veracruz, the largest producer nationwide [2].

Phythopatogenic fungi are responsible for most of the deterioration in fruit and vegetables [3]. Most of these fungi attack the fruit in the field, and they have the capacity to survive in the soil and among the different plant cycles, being able to survive as mycelium or in any of their different types of spores, dispersing through leaves, fruit, and contaminated equipment, as well as by the wind [3,4]. Regarding chayote fruit, its high moisture content in fresh state, high transpiration, and the use of plastic bags favor its wilting and the reduction in its shelf life. During postharvest, because chayote fruit have a thin epidermis, they are very susceptible to damage caused by rubbing, compression, and impact, which is why they are mainly affected by diseases, such as bladder or blister (*Colletotrichum gloeosporioides*), anthracnose (*C. orbiculare*), white mold (*Phytophthora capsici*), red-purple mold (*Fusarium* spp.), and sour rot (*Geotrichum* sp.) [4].

Although the use of synthetic fungicides helps to extend the shelf life of these products, their drawbacks, such as toxicity to humans, export detentions due to residues in the product, damage to the environment, and detrimental effects on beneficial organisms [5], generate the need to look for natural options that are biodegradable and safe for the environment, as well as safe for human consumption and that do not generate resistant strains of pathogens.

In different studies around the world, secondary metabolites with biological activity have been found to be located in plants such as flavonoids, saponin concentrates, eriodictyol, citronellal, citronellol, tannins, phenols, and triterpenes, to name a few [6]. Because of this, plant extracts and essential oils, which have shown a fungistatic or fungicidal effect, have been evaluated in the control of postharvest fungi in fruit and vegetables [7]. Examples include the following: extracts of cinnamon (*Cinnamomum zeylanicum* Blume), guava (*Psidium guajava* L.), and lemon grass (*Cymbopogon citratus* (DC.) Stapf) against *C. gloeosporoides* in vitro [8]; extracts of Syrian oregano (*Origanum syriacum*), olivarda (*Inula viscosa*), and horse mint (*Mentha longifolia*) against *Fusarium oxysporum* in vitro [9]; essential oils of oregano (*Origanum vulgare*), thyme (*Thymus capitatus*), díctamo (*Origanum dictamnus*), marjoram (*Origanum majorana*), lavender (*Lavandula angustifolia*), and rosemary (*Rosmarinus officinalis*) against *Fusarium* sp. [10]. Although extracts of cinnamon have a great fungicidal action against pathogenic microorganisms due to cinnamaldehyde content, some other biocontrol agents could be suitable for application in postharvest.

On the other hand, it is known that products derived from neem (*Azadarichta indica*) affect insects, mites, Gram-positive bacteria, nematodes, snails, and fungi such as *Aspergillus* sp. [11,12]. However, its effect on phytopathogenic fungi that cause diseases in fruit and vegetable products has not been studied.

Finally, although the microbial and fungicidal properties of black sapote (*Diospyros digyna*) have been evaluated on pathogens that cause diseases in humans, no extracts of *D. digyna* leaves, fruit, or seeds have been evaluated in the control of postharvest diseases of fruit and vegetables.

Based on the above, the aim of this study was to evaluate the in vitro antifungal activity of cinnamon essential oil, neem oil, and black sapote extract on fungi that cause postharvest diseases in chayote fruit.

## 2. Results

### 2.1. Isolation and Identification of Fungal Strains

**Figure 1:**
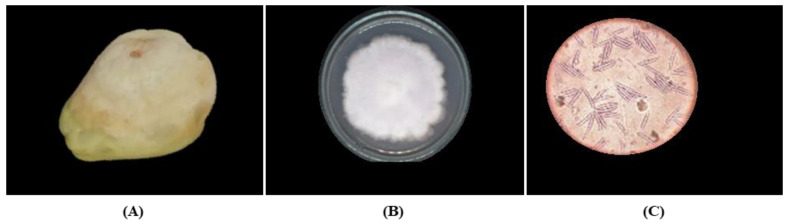
Symptomatology and colony characteristics of *F. oxysporum* isolated from chayote fruit. (**A**) Chayote fruit infected by *F. oxysporum* with presence of white mycelium and rot, (**B**) isolation of *F. oxysporum* from chayote fruit, and (**C**) macroconidia morphologies of *F. oxysporum* observed via light microscopy 40×.

**Figure 2:**
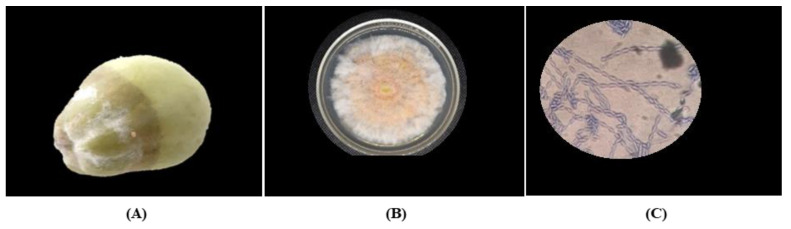
Symptomatology and colony characteristics of *F. solani* isolated from chayote fruit. (**A**) Chayote fruit infected by *F. solani* with presence of white mycelium and rot, (**B**) isolation of *F. solani* from chayote fruit, and (**C**) monofialides morphologies of *F. solani* observed via light microscopy 40×.

**Figure 3:**
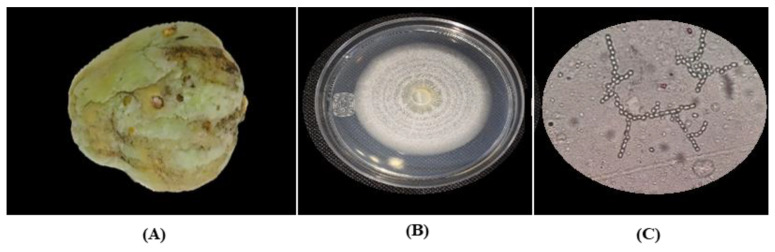
Symptomatology and colony characteristics of *Geotrichum* sp. isolated from chayote fruit. (**A**) Chayote fruit infected by *Geotrichum* sp. with presence of acid rot, (**B**) isolation of *Geotrichum* sp. from chayote fruit, and (**C**) colony morphologies of *Geotrichum* sp. observed via light microscopy 40×.

**Figure 4:**
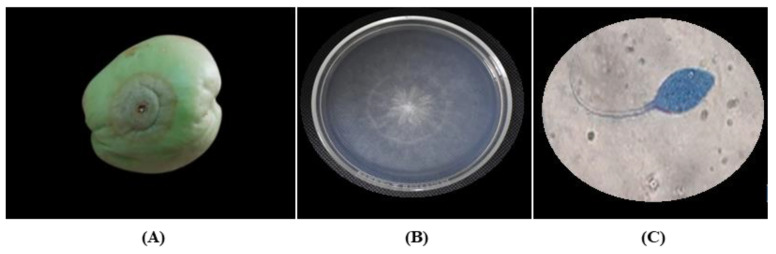
Symptomatology and colony characteristics of *P. capsici* isolated from chayote fruit. (**A**) Chayote fruit infected by *P. capsici* with presence of white mold, (**B**) isolation of *P. capsici* from chayote fruit, and (**C**) sporangium morphologies of *P. capsici* observed via light microscopy 40×.

A total of four fungi were isolated from chayote fruit: *F. oxysporum* (Figure 1), *F. solani* (Figure 2), *Geotrichum* sp. (Figure 3), and *P. capsici* (Figure 4). The symptom present in the chayote fruit infected by *Fusarium* spp. was the presence of spongy mycelium of different shades of white-pinkish or violet (Figure 1A and Figure 2A). *F. oxysporum* presented colonies with abundant aerial mycelium, similar to cotton and white in color, which turned purple after a few days (Figure 1B). On the other hand, *F. solani* presented an extensive mycelium, similar to cotton, and the color of the colony varied from yellow to pink (Figure 2B). In the microscopic observation different types of spores could be seen. For *F. oxysporum*, these were comma-shaped unicellular microconidia or canoe-shaped macroconidia with three to five septa (Figure 1C). On the other hand, the mycelium of *F. solani* was septate, with monifialides with a chain (Figure 2C).

The symptom observed in the chayote fruit infected by *Geotrichum* sp. was the presence of acid rot (Figure 3A). *Geotrichum* sp. developed white–cream-colored colonies, which over time turned to a shade of a pink hue, and also presented a sparse mycelium with ring formations (Figure 3B). In the microscopic observation, the structures of the hyphae were septate, with classifications of rectangular to cylindrical arthroconidia, and with the formation of chains or unicellular arthroconidia (Figure 3C).

The present symptom in the chayote fruit infected by *P. capsici* was ring-shaped watery spots on the epicarp that enlarged with time, and which were covered with mycelium, which also developed internally (Figure 4A). *P. capsici* presented colonies with transparent white flower- or star-shaped characteristics with little aerial mycelium (Figure 4B). In the microscopic observation, sporangia in head, papilate sporangia, and sporangia with long pedicel and oospores were observed (Figure 4C).

### 2.2. Effectiveness of Plant Extracts

Cinnamon essential oil, neem oil, and black sapote fruit extract were evaluated at 300, 350, and 400 ppm on the in vitro growth of the following fungi isolated from chayote fruit: *F. oxysporum*, *F. solani*, *Geotrichum* sp., and *P. capsici*. Cinnamon essential oil inhibited 100% of fungal growth at all concentrations used (Table 1, see Supplemental Figures S1–S36), and is therefore considered to have a fungicidal effect. On the other hand, neem oil and sapote extract inhibited fungal mycelial growth compared to the control, exhibiting a fungistatic effect (Table 1).

The effectiveness of neem oil was different for each fungal species, being higher for *P. capsici,* which was inhibited by 56.09% at 350 ppm, and *F. solani*, which was inhibited by 42.29% at 400 ppm, while the genus *Geotrichum* was inhibited by 22.33% at 350 ppm, and *F. oxysporum* was inhibited by 27.82% at 400 ppm. On the other hand, the black sapote extract presented an effectiveness proportional to its concentration on all the microorganisms evaluated, and this was similar (21.95–28.61%) on all fungi at a concentration of 400 ppm. However, black sapote extract at 400 ppm was more effective than neem oil against *Geotrichum* (28.61 and 20.96%, respectively). In addition, *F. oxysporum* and *F. solani* developed a less-dense mycelium in the black sapote extract treatments compared to control treatment.

**Figure 5:**
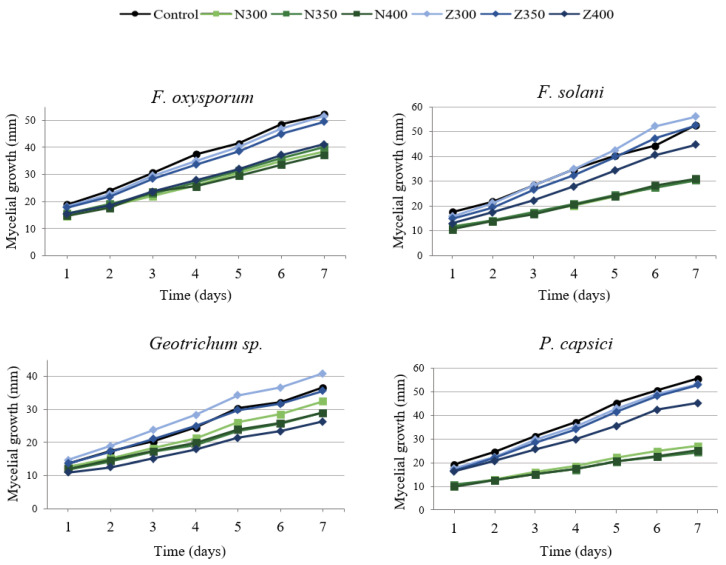
Effect of neem oil (N300, N350, and N400) and sapote extract (Z300, Z350, and Z400) at 300, 350, and 400 ppm on mycelial growth of fungi isolated from chayote fruit and an absolute control.

Figure 5 shows the daily mycelial growth of fungi treated with neem oil and black sapote extract. During the 7 days of incubation, the fungi showed constant mycelial growth. In general, the neem oil treatments showed less mycelial growth over time compared to the control. On the other hand, fungi treated with sapote extract at 400 ppm showed less mycelial growth compared to the control during the time of the experiment.

## 3. Discussion

Plants contain secondary metabolites that can be useful in the control of phytopathogenic fungi during postharvest. These compounds can be isolated from plant tissue or applied through plant extracts or essential oils made from different plant organs [7]. Several studies have demonstrated the antifungal activity of dozens of bioactive compounds present in plants and their effectiveness depends on their concentrations and the pathogen in question [7,13].

Essential oils are often particularly useful for their antimicrobial properties. They have the ability to penetrate the cell membranes of microorganisms, due to their hydrophobic characteristic. By affecting the cell wall, they increase permeability and generate the leakage of ions and other materials from the cell, causing cell death [14,15].

Several studies have found fungicidal and fungistatic effects of cinnamon extracts and essential oils, which coincide with the results obtained in the present study with fungi isolated from chayote fruit. Antifungal activity has been found in the cinnamic aldehyde of cinnamon [16], while in this work cinnamon essential oil allowed total inhibition of the mycelial development of phytopathogenic fungi, as well as the inhibition of the growth of *Fusarium proliferatum* [17,18]. The cinnamon extract used demonstrated its antifungal potential, indicating the possibility of its use in the in vitro control of phytopathogenic fungi in chayote fruit. Some compounds that may be related to the antifungal effects of cinnamon are cinnamaldehyde, kaempferol, and cinnamic alcohol, among others [19].

In this study, *F. oxysporum*, *F. solani*, *Geotrichum* sp., and *P. capsici*, which caused diseases such as reddish-purple mold, acid rot, and white mold, were isolated. Ochoa-Fuentes et al. [20] showed mycelial growth inhibition of 31.81% on *F. culmorum*, 43.15% on *F. oxysporum*, and 45.58% on *F. solani* (45.58%) by applying a methanolic extract of cinnamon at 300 ppm, while an aqueous extract of cinnamon at a concentration of 500 ppm presented antifungal activity against *F. oxysporum*, *Alternaria alternata*, and *Geotrichum candidum*, among others [19]. In addition, Ramírez-González et al. [21] studied the effect of cinnamon oil at 0.1% (*v/v*) on fungi isolated from papaya, which had a fungicidal effect against *A. solani* and *C. gloesporioides*, while conidial formation was inhibited, and mycelial growth was controlled on *F. oxysporum*.

Pazmiño-Miranda et al. [22] demonstrated the effectiveness of a hydroethanolic cinnamon extract at a concentration of 15 mL L^−1^ against *Botrytis cinerea*, reducing the incidence and severity of gray rot in strawberries. Similarly, Kowalska et al. [23] were able to inhibit gray mold (*Botrytis cinerea*) in tomato plants by 81.4% with an aqueous extract of cinnamon at 1% (*w/v*); additionally, the extract stimulated plant growth.

In relation to neem, more than 300 secondary metabolites have been found. Among them, one third are limonoids with known biological effects, such as nimbin, nimbidin, and azadirachtin; the last has been considered the main component with biological activity derived from neem [24,25]. Alkaloids, essential oils, polyphenols, tannins, and saponins have also been found in an ethanolic extract of neem leaves [26].

In addition, in vitro and in vivo studies have been conducted with neem extracts applied to different phytopathogenic fungi. In such research, methanolic extract of neem seeds at a concentration of 0.0300% inhibited the development of *C. gloeosporioides* mycelium and at 0.1200% it controlled germination significantly compared to the control [27], while neem oil inhibited mycelial growth of *Schizophyllum commune*, *F. oxysporum*, *F. proliferatum*, *Coniophora puteana*, and *Alternaria alternata*; in this case, treatments with concentrations greater than 2% were the most effective [28]. In addition, Bolivar et al. [26] applied a 2.5% ethanolic extract of neem to mango fruit inoculated with *C. gloeosporioides* and observed that the diameter of the lesions decreased by 19.9% compared to the control. Lal et al. [29] studied two potato cultivars over two years, to which they applied 6% neem oil, 1% *Pseudomonas fluorescens*, and 2% *Trichoderma viride*, and observed that neem oil reduced the incidence of the disease caused by *Phytophthora infestans* more effectively than the other treatments. In addition, toxicological tests are still necessary to expand the coverage of the safety of these substances concerning human health.

Results of the aforementioned experiments confirm the antifungal activity of the essential oil of neem against phytopathogenic fungi isolated from chayote fruit. In general, it has been shown that the effectiveness of neem-derived extracts can vary depending on the maturity of the fruit, the genetic variety, the extraction method, the geographical area, and the purity of the oil [20,30,31].

Finally, regarding the black sapote extracts, although little research has been conducted to evaluate their antifungal activity, compounds with biological activity have been found in various species of the *Diospyros* genus, such as kaempferol, quercetin, gallic acid, plumbagin, diospyrin, and cinnamaldehyde, among others [32,33,34]. Dzoyem et al. [35] evaluated an extract from the stem bark of *Diospyros crassiflora* in methanol/dichloromethane (1:1) and found that it inhibited the growth of *Candida albicans*, *Candida glabrata, Candida krusei, Candida tropicalis*, *Cryptococcus neoformans*, *Aspergillus niger*, *Aspergillus flavus*, *Alternaria* sp., *Cladosporium* sp., *Geotrichum candidum*, *Fusarium* sp., and *Penicillium* sp., with minimum inhibitory concentration values of 12.5–25 mg mL^−1^. In another study, 50 plant extracts were evaluated against *Aspergillus niger* and *Rhizopus stolonifer*; in this case, the ethanolic extract of black sapote tree bark at 156 µg mL^−1^ showed a significant effect against *A. niger*, but had no effect against *Rhizopus stolonifer* [36].

Subsequently, Ramírez-Briones et al. [37] studied the effect of extracts of *Diospyros rekoi* and *Diospyros dígyna* leaves against known phytopathogenic species and found no antifungal activity of the extracts. In this regard, the authors suggest the possibility that the polar solvents used for the preparation of the extracts did not allow the extraction of compounds such as naphthoquinones, which have greater biocidal capacity; on the other hand, the extracts presented phenolic compounds in their composition, known to be effective against fungi and yeasts, so it is important to carry out further studies on the subject.

In the present study, the concentration used for the sapote extract and neem oil had a fungistatic effect, so the possibility that the bioactive compounds volatilize or lose their effectiveness due to decay, denaturation, or inactivation over time can be considered [38]. However, it was observed that neem essential oil at the concentrations evaluated efficiently inhibited the development of *P. capsici* (more than 50% inhibition), and that cinnamon essential oil was effective against all phytopathogens; consequently, these could be incorporated into coating formulations for application to postharvest chayote fruit, with an integrated management approach due to their antifungal properties.

## 4. Materials and Methods

### 4.1. Plant Material

Chayote fruit were collected at horticultural maturity in a commercial orchard in the town of Coxcontla, municipality of Huatusco, Veracruz, Mexico. Fruit with symptoms of chayote fruit diseases characteristic of the postharvest period (bladder or blister, anthracnose, white mold, and reddish-purple mold) were selected and placed in sterilized paper bags and later in plastic bags. The chayote fruit were transported in a cooler and processed the same day at the Dairy Laboratory of the Postgraduate College Campus Cordoba.

### 4.2. Isolation and Identification of Fungal Strains

Twenty fruits with diseased and healthy tissue were selected and washed with distilled water and detergent, cut into pieces of approximately 125 mm^3^, and disinfected with 2% commercial sodium hypochlorite for 2 min. They were rinsed with sterile distilled water and placed on sterile adsorbent paper to remove any remaining moisture. Then, 4 pieces per Petri dish were placed in a potato dextrose agar (PDA) culture medium (Dibico, Mexico City, Mexico) and kept at 27 ± 1 °C for 15 days in an incubator (Riossa, ECF—82.D, Monterrey, Nuevo Leon, Mexico).

For the purification of fungi, the hyphal tip technique was applied in the PDA culture medium; the tips were incubated at 27 ± 1 °C for 5 days [39]. Fungal strains isolated from chayote fruit were preserved in distilled water (70%)–glycerol (30%) at −20 °C. The microorganisms were preserved in a Sabouraud dextrose agar culture medium (Dibico, Mexico City, Mexico) [30,40,41].

For morphological characterization, semi-permanent preparations were made with cotton blue and observed under a compound microscope (Carl Zeiss Microlmaging GmbH 37081, Gottingen, Germany). Morphological identification was made using the taxonomic keys of Barnett and Hunter [42]; Arévalo-Galarza et al. [4]; Olguín-Hernández et al. [43]; Olguín-Hernández et al. [44]; Olguín-Hernández et al. [45]; and Romero-Velázquez et al. [39].

### 4.3. Plant Extracts

Cinnamon (*Cinnamomum zeylanicum*) essential oil was obtained from Aceites y Esencias S. A. (Mexico City, Mexico) and neem (*Azadirachta indica*) oil from Geoquímica industrial IBCER, S. A. de C. V. (Los Mochis, Sinaloa, Mexico). The sapote (*Diospyros digyna*) extract was made with fruit collected at physiological maturity in the community of Manuel León, Veracruz, Mexico. The fruit were cleaned with 96° alcohol, chopped, and liquefied for two minutes in a blender (Oster, Boca Ratón, FL, USA) with a sterilized glass jar, and filtered with sterile gauze; the extract was then added to the PDA culture medium.

### 4.4. In Vitro Evaluation of Plant Extracts on Postharvest Fungi

The evaluation of the in vitro effect of plant extracts on fungi isolated from chayote fruit was carried out using the culture medium poisoning method [46]. The extracts were added at concentrations of 300, 350, and 400 ppm, each in Petri dishes with a PDA culture medium before solidification; an absolute control without plant extract was also included. When the culture medium solidified, the corresponding strains were added to form a 5 mm diameter disk (10 μL of a fungal suspension was inoculated into the medium, concentration 1 × 10^8^ spores mL^−1^) and left to incubate (Riossa, ECF—82, Monterrey, Nuevo Leon, Mexico) at 27 ± 1 °C; mycelial growth was measured every 24 h for 7 days. Percentage inhibition of daily mycelial growth was calculated using Equation (1) [47].

(1) $$\%\;inhibition=\frac{Ccd-Tcd}{Ccd}\times 100$$

where:

*Ccd*: control colony diameter.

*Tcd*: treatment colony diameter.

### 4.5. Statistical Analysis

A randomized complete block design with repeated measures was used to evaluate the effect of plant extracts at 0, 300, 350, and 400 ppm on fungal growth for seven days. The experiment consisted of two blocks and 6 repetitions were carried out in each block. Statistical analysis was performed using the GLIMMIX procedure of SAS version 9.4. An AR(1) covariance structure and LSD mean comparison at a 5% confidence level were used. The model is described below:

$$y_{ijkl}=\mu +b_{k}+\alpha_{i}+\omega_{ikl}+\tau_{j}+\gamma_{ij}+\varepsilon_{ijkl}$$

where $y_{ijkl}$ is the percentage inhibition of fungal growth, μ is the overall mean, $b_{k}$ is the random effect due to block $\left\{b_{k}\;\sim \;N\;\left(0,\;\sigma_{b}^{2}\right)\right\}$, $\alpha_{i}$ is the fixed effect due to oil concentration, $\omega_{ikl}$ is the random effect due to block-repetition $\left\{\omega_{ikl}\;\sim \;N\;\left(0,\;\sigma_{\omega }^{2}\right)\right\}$, $\tau_{j}$ is the fixed effect due to measurement time, $\gamma_{ij}$ is the effect of oil concentration interaction with measurement time, and $\varepsilon_{ijkl}$ is the experimental error $\left\{\varepsilon_{ijkl}\;\sim \;N\;\left(0,\;\sigma^{2}\right)\right\}$.

## 5. Conclusions

Commercial agriculture depends mainly on the application of chemical fungicides to protect crop plants against fungal pathogens. However, the emergence of resistant strains of fungal phytopathogens makes plant fungal diseases increasingly challenging to treat. Accordingly, the development of healthy, non-toxic alternate approaches to chemical fungicides, such as the botanical fungicides evaluated in this study, is very helpful in the control of plant fungal infections. Different phytopathogen fungi species (*F. oxysporum*, *F. solani*, *Goetrichum* sp., and *Phytophthora capsici*) isolated from chayote fruit were inhibited by plant extracts. Cinnamon essential oil completely inhibited fungal growth. Therefore, it is an effective option for inhibiting the growth of fungi that cause common fungal diseases in chayote fruit during postharvest. On the other hand, neem oil presented greater inhibition capacity against *F. solani* and *Phytophthora capsica*, and sapote extract was more effective against the genus *Geotrichum*, so both can be alternatives to control these strains. However, further research is needed to evaluate the effect of the extracts in in vivo studies in order to determine optimal concentrations. In addition, different types of extracts need to be evaluated.

## Figures and Tables

**Table 1:** Cinnamon oil, black sapote extract, and neem oil treatments applied to fungi isolated from chayote fruit and their effect on inhibition of mycelial growth after 7 days of incubation at 27 °C.

Treatment	Concentration (ppm)	*Fusarium oxysporum*	*Fusarium solani*	*Geotrichum* sp.	*Phytophthora capsici*
		(% Inhibition)			
Cinnamon oil	300	100.00 a	100.00 a	100.00 a	100.00 a
	350	100.00 a	100.00 a	100.00 a	100.00 a
	400	100.00 a	100.00 a	100.00 a	100.00 a
Sapote extract	300	4.70 hi	2.71 i	3.94 hi	7.88 ghi
	350	7.75 ghi	8.17 ghi	7.67 ghi	10.19 gh
	400	26.07 def	21.95 ef	28.61 e	24.28 def
Neem oil	300	24.69 def	39.50 c	13.34 g	51.87 b
	350	22.56 def	40.70 c	22.33 def	56.09 b
	400	27.82 de	42.29 c	20.96 f	53.31 b

Source: author—made. Different letters indicate significant differences (*p* < 0.05).

## Data Availability

Not applicable.

## Supplementary Materials

The following supporting information can be downloaded at: https://www.mdpi.com/article/10.3390/plants12091800/s1.
