# Supplementary material for: Plant Extracts Control In Vitro Growth of Disease-Causing Fungi in Chayote
Source: Plants (Basel). 2023 Apr 27;12(9):1800. doi: 10.3390/plants12091800 (PMC10180525; doi:10.3390/plants12091800)
Supplement: Supplementary file 1 [file plants-12-01800-s001.zip › plants-2267963-supplementary.pdf]

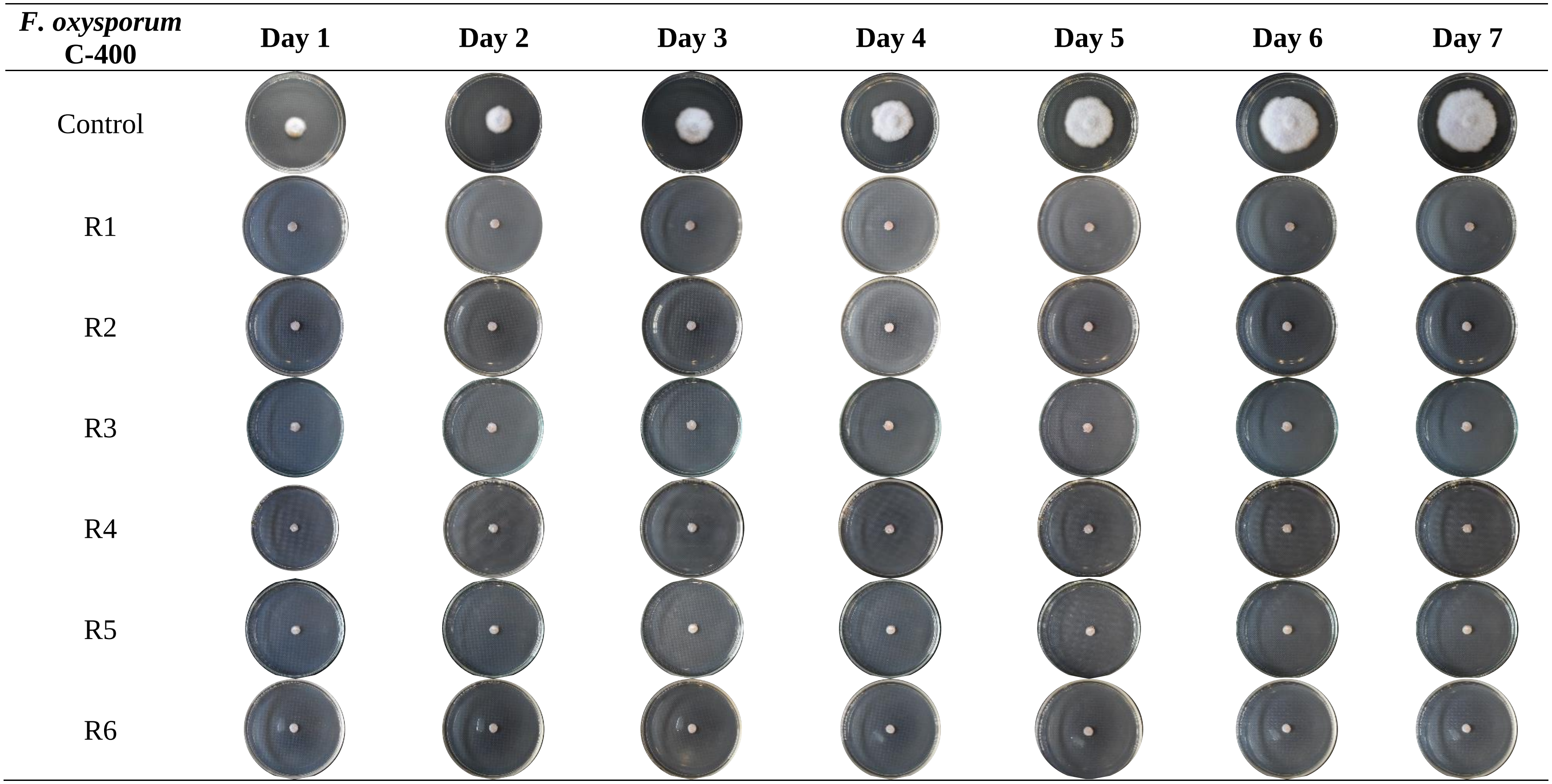

Figure S1. Effect on fungal growth of *Fusarium oxysporum* after treatment with 400 ppm of cinnamon oil. R1-R6: repetitions

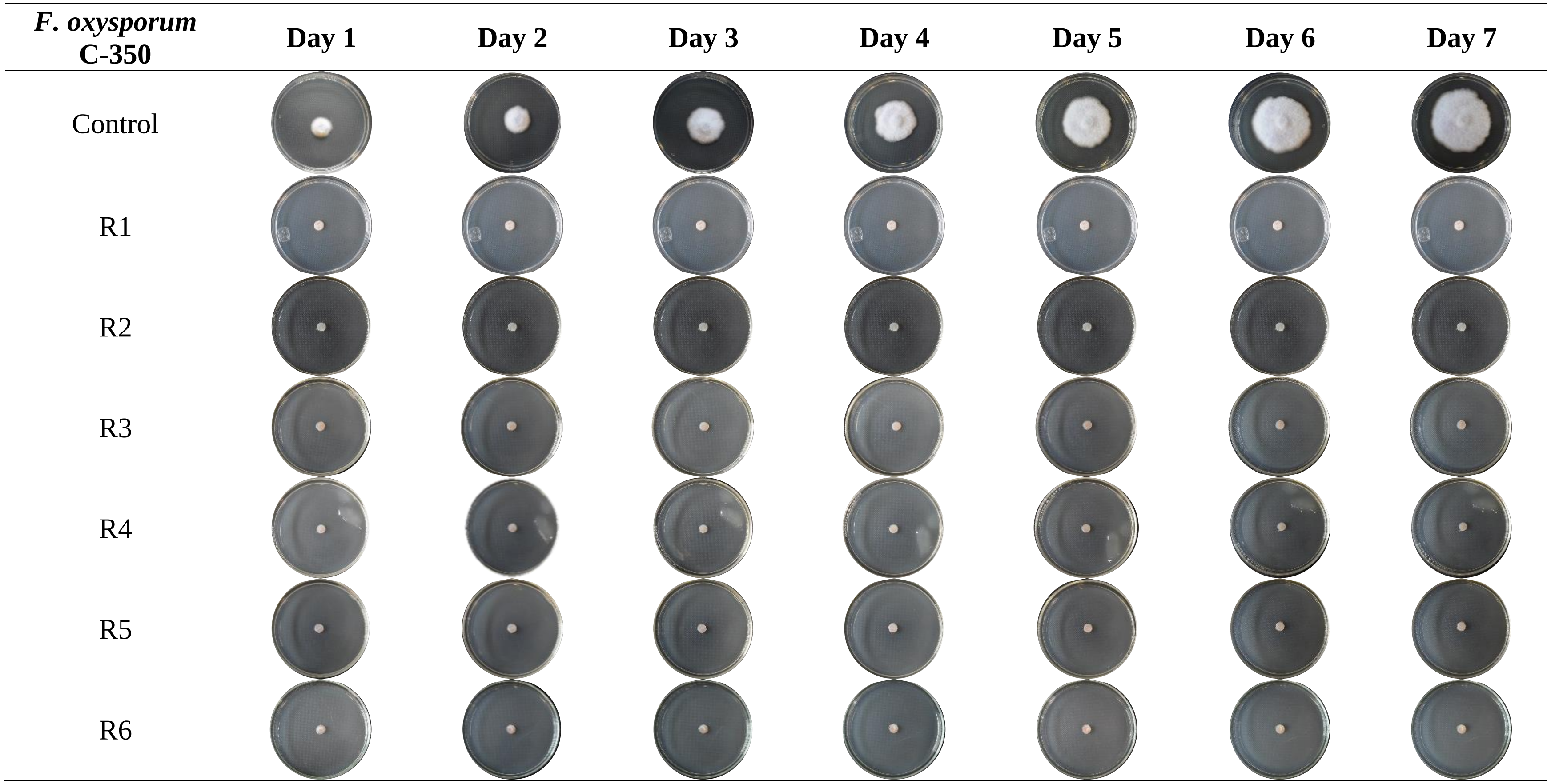

Figure S2. Effect on fungal growth of *Fusarium oxysporum* after treatment with 350 ppm of cinnamon oil. R1-R6: repetitions

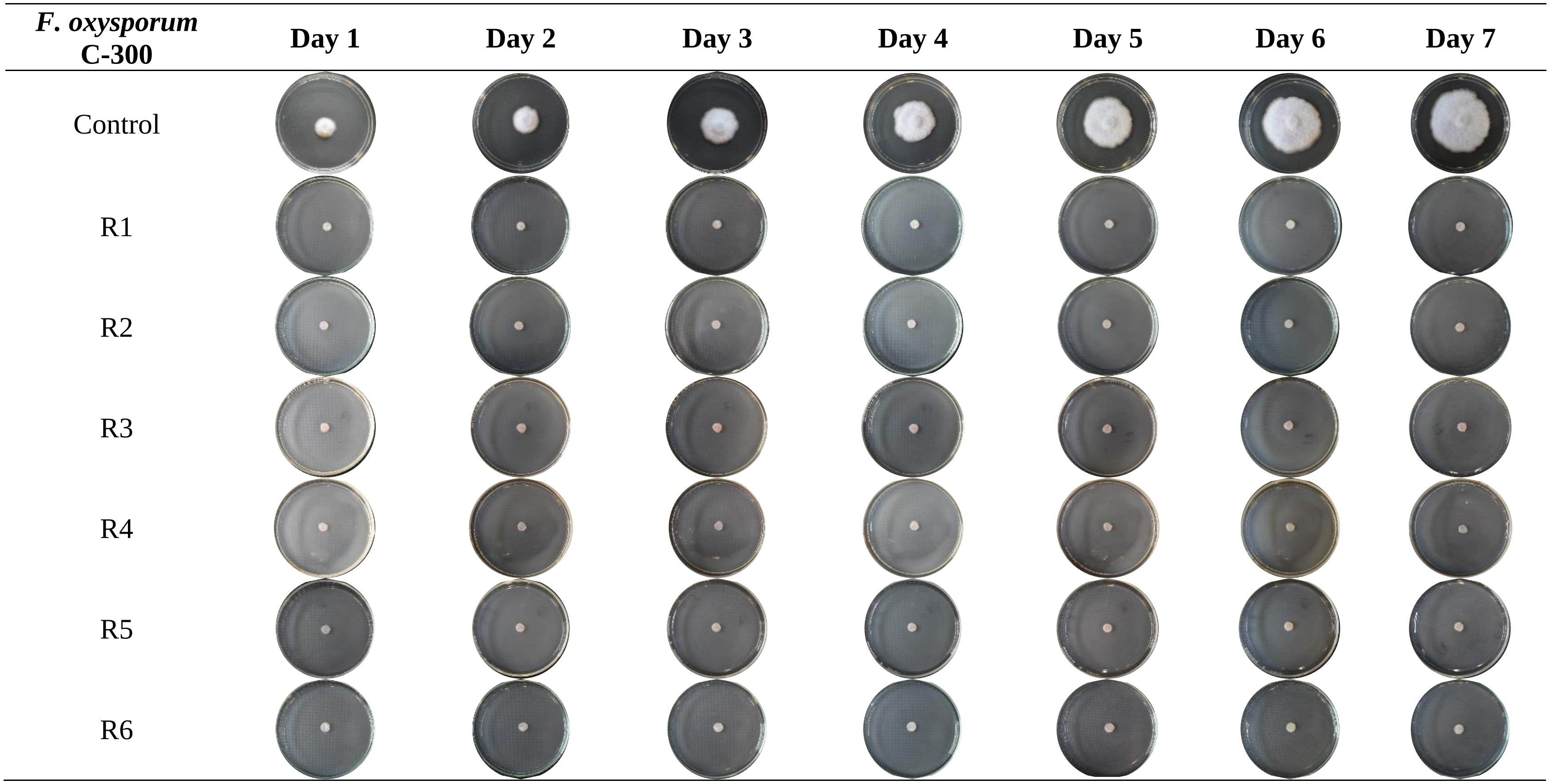

Figure S3. Effect on fungal growth of *Fusarium oxysporum* after treatment with 300 ppm of cinnamon oil. R1-R6: repetitions

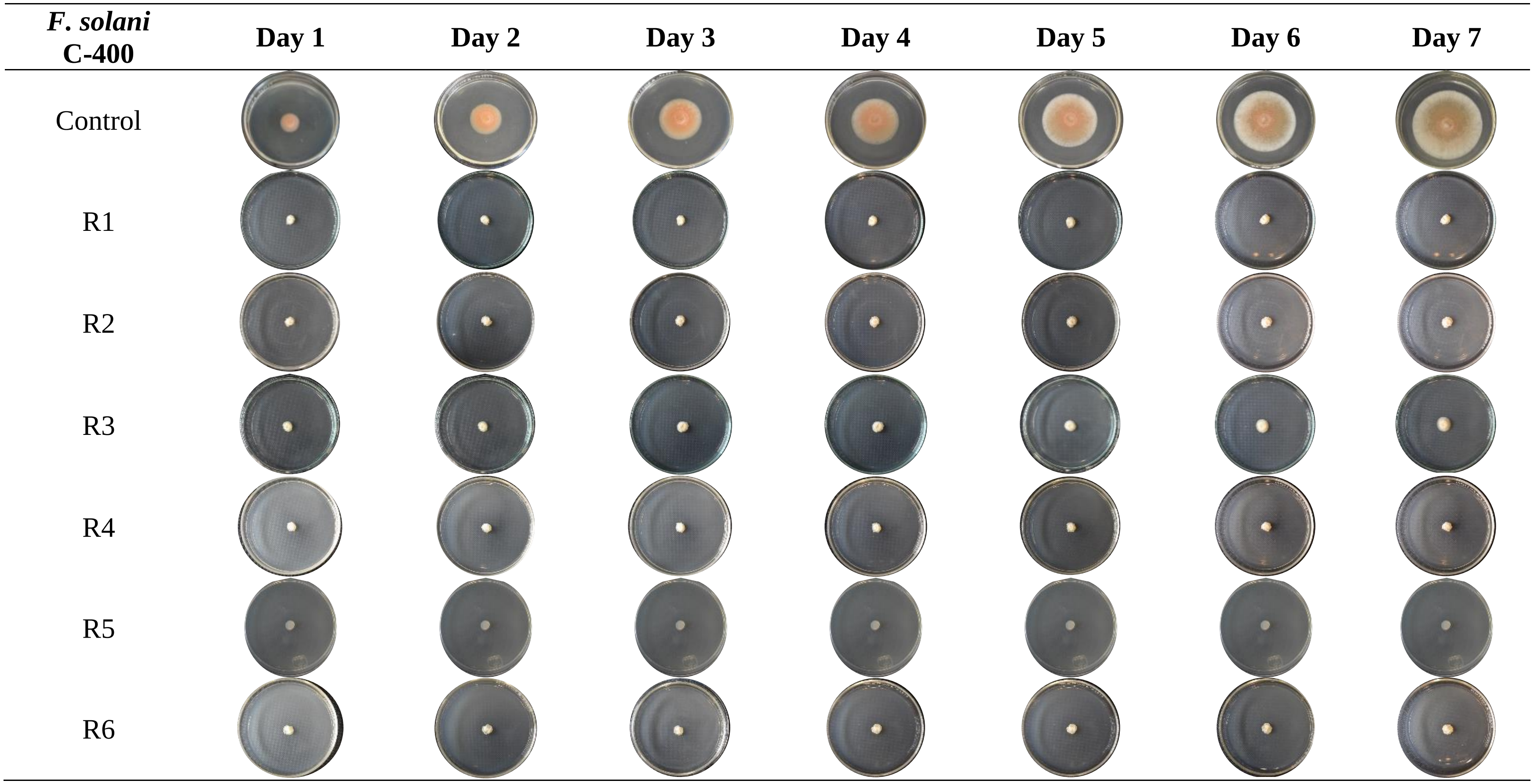

Figure S4. Effect on fungal growth of *Fusarium solani* after treatment with 400 ppm of cinnamon oil. R1-R6: repetitions

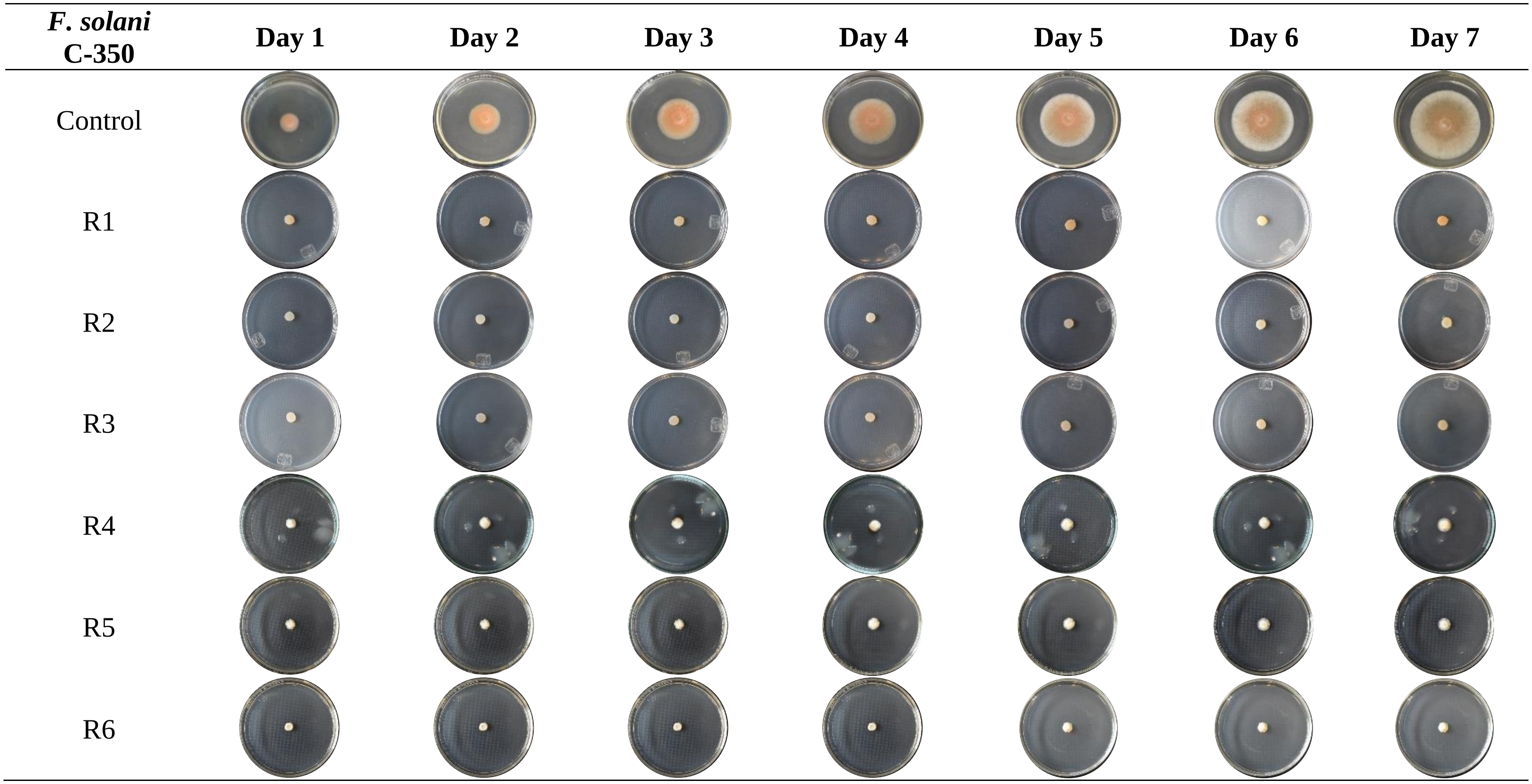

Figure S5. Effect on fungal growth of *Fusarium solani* after treatment with 350 ppm of cinnamon oil. R1-R6: repetitions

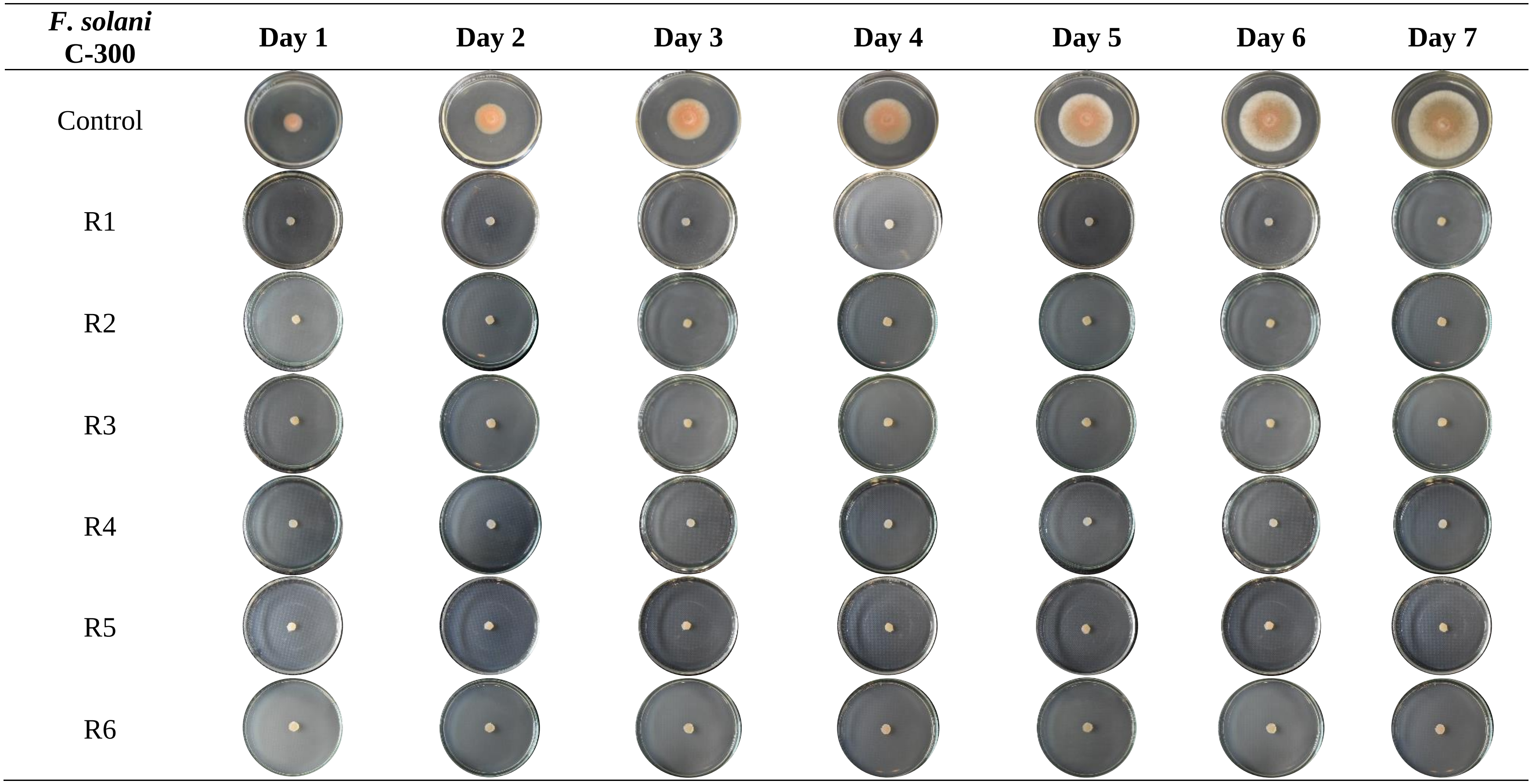

Figure S6. Effect on fungal growth of *Fusarium solani* after treatment with 300 ppm of cinnamon oil. R1-R6: repetitions

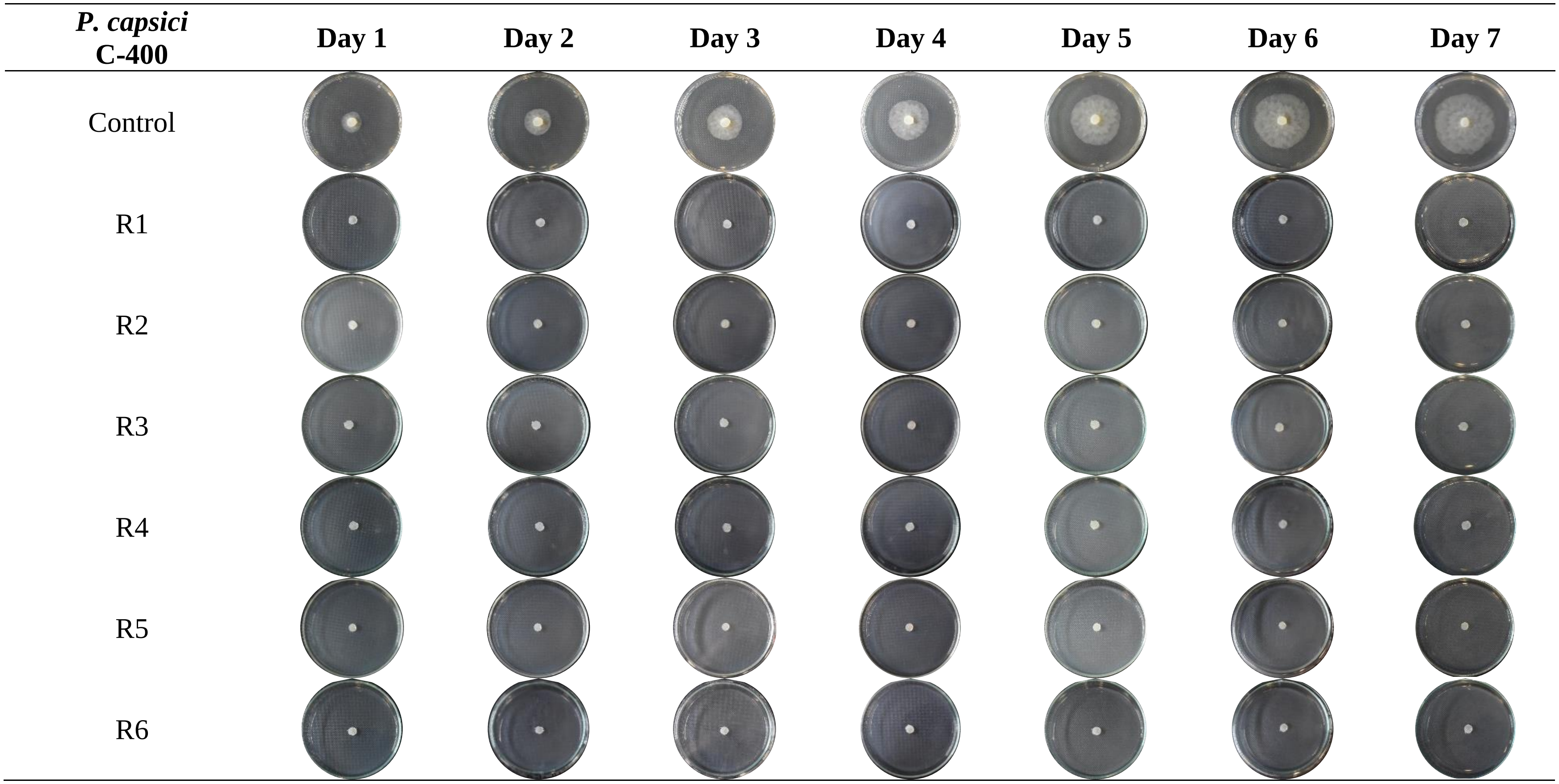

Figure S7. Effect on fungal growth of *Phytophthora capsici* after treatment with 400 ppm of cinnamon oil. R1-R6: repetitions

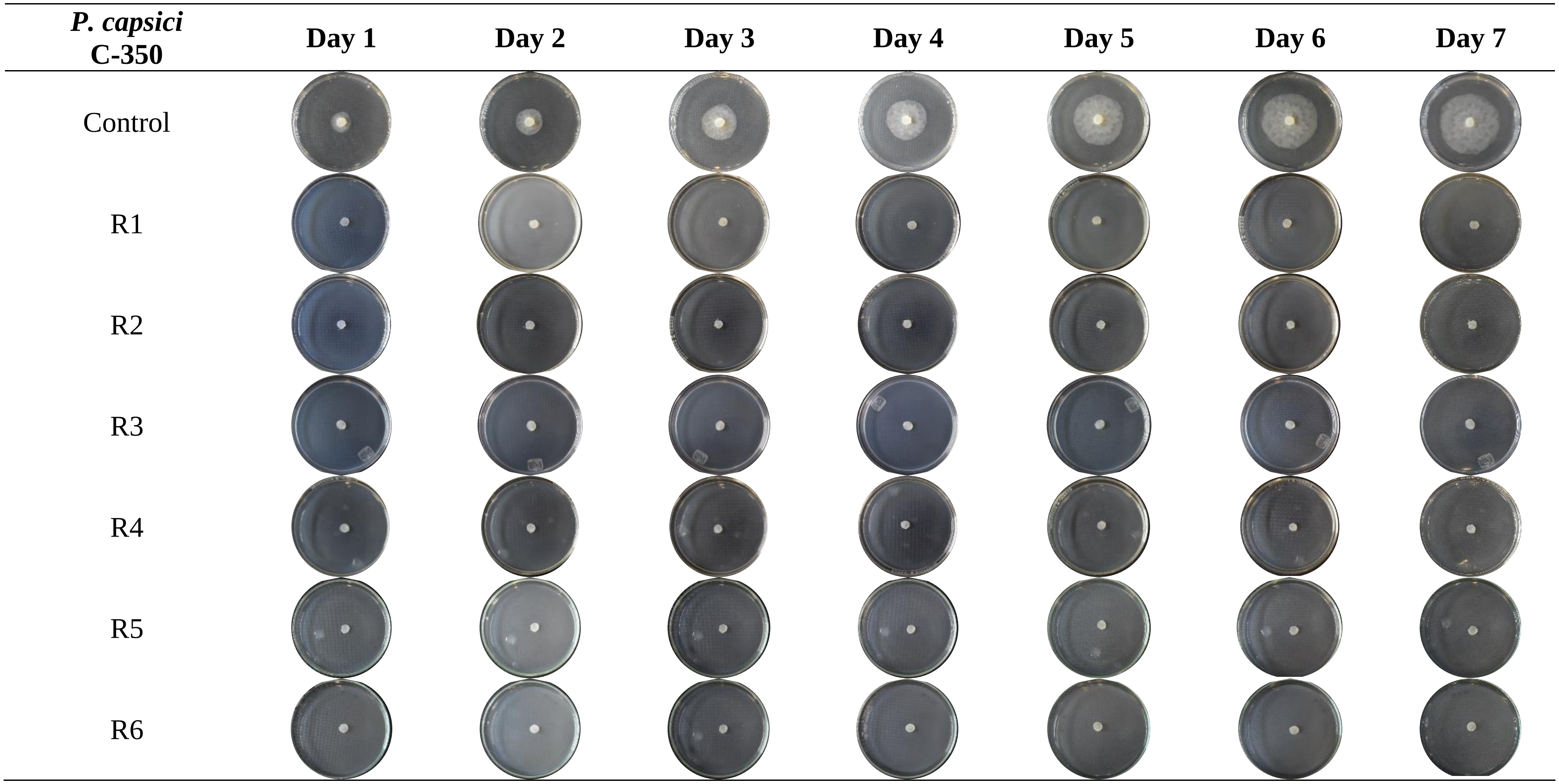

Figure S8. Effect on fungal growth of *Phytophthora capsici* after treatment with 350 ppm of cinnamon oil. R1-R6: repetitions

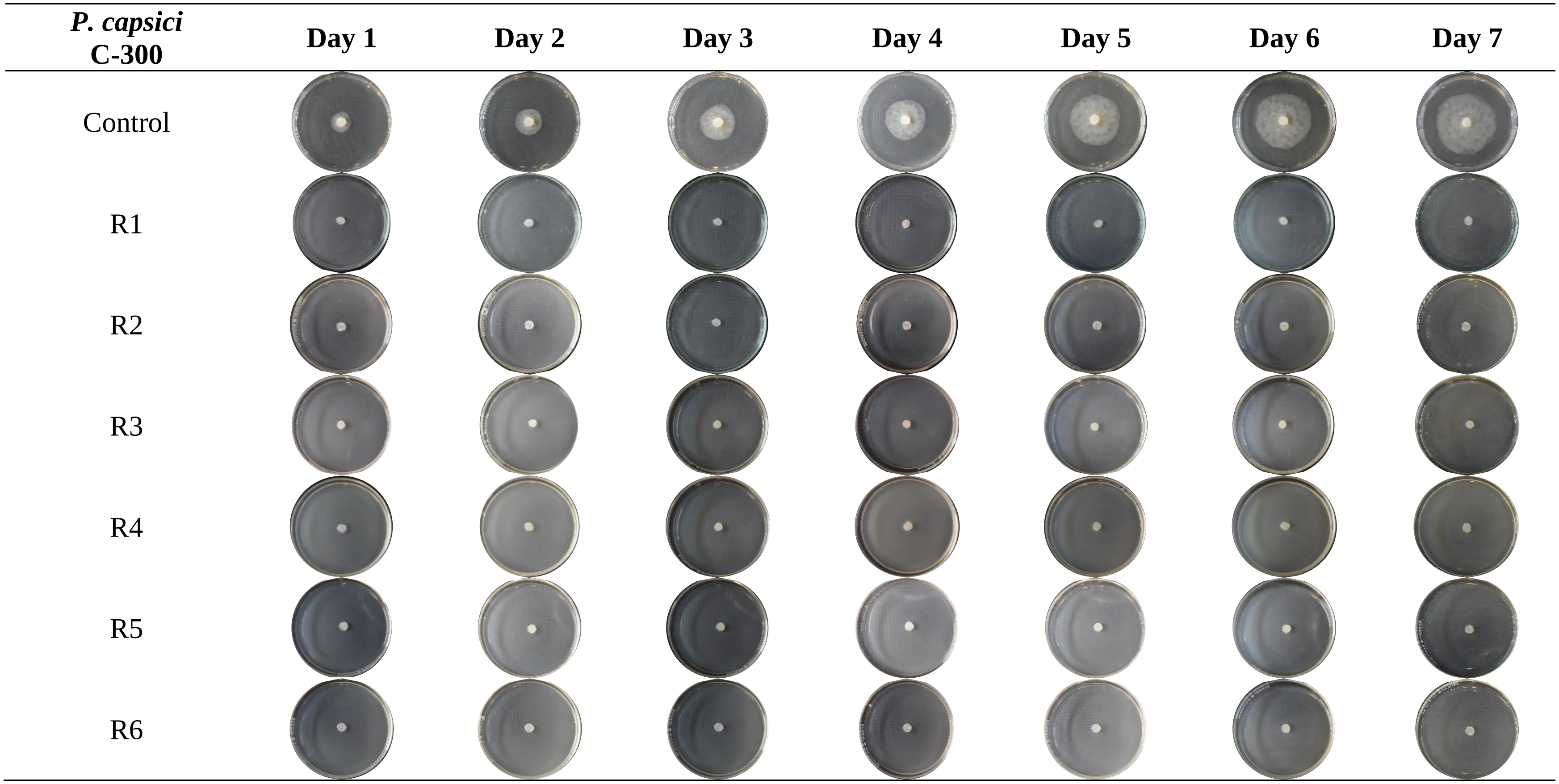

Figure S9. Effect on fungal growth of *Phytophthora capsici* after treatment with 300 ppm of cinnamon oil. R1-R6: repetitions

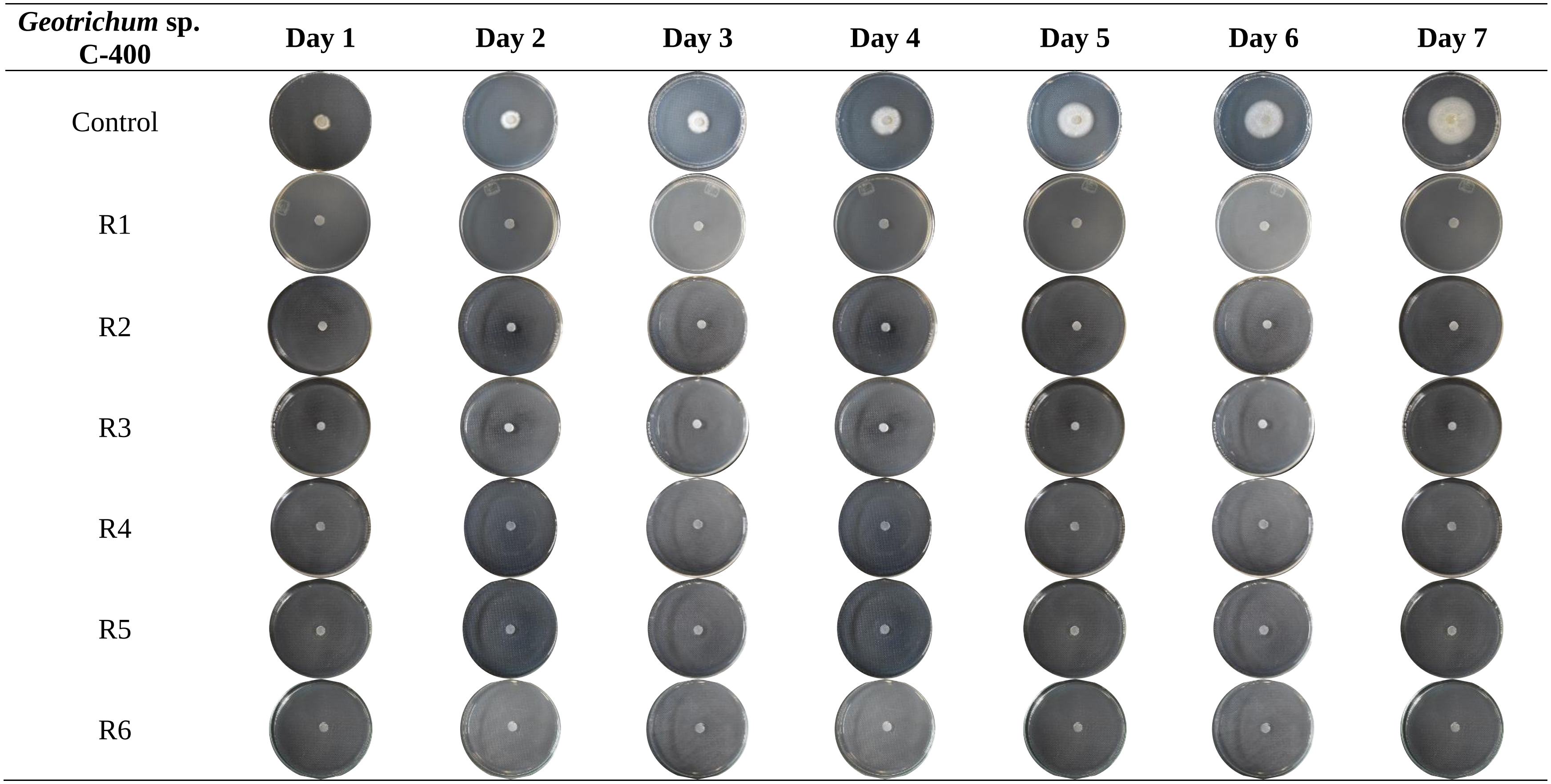

Figure S10. Effect on fungal growth of *Geotrichum* sp. after treatment with 400 ppm of cinnamon oil. R1-R6: repetitions

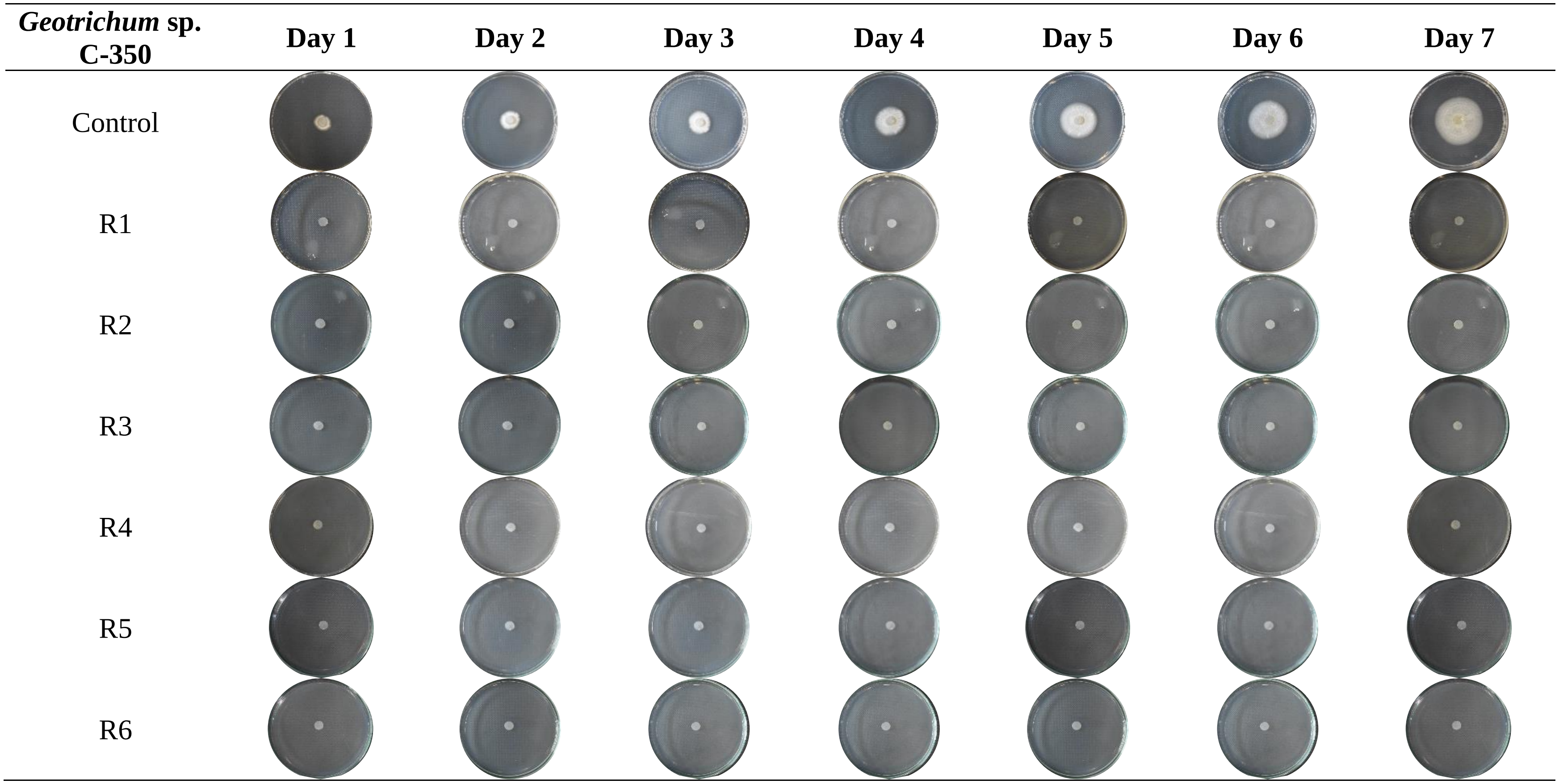

Figure S11. Effect on fungal growth of *Geotrichum* sp. after treatment with 350 ppm of cinnamon oil. R1-R6: repetitions

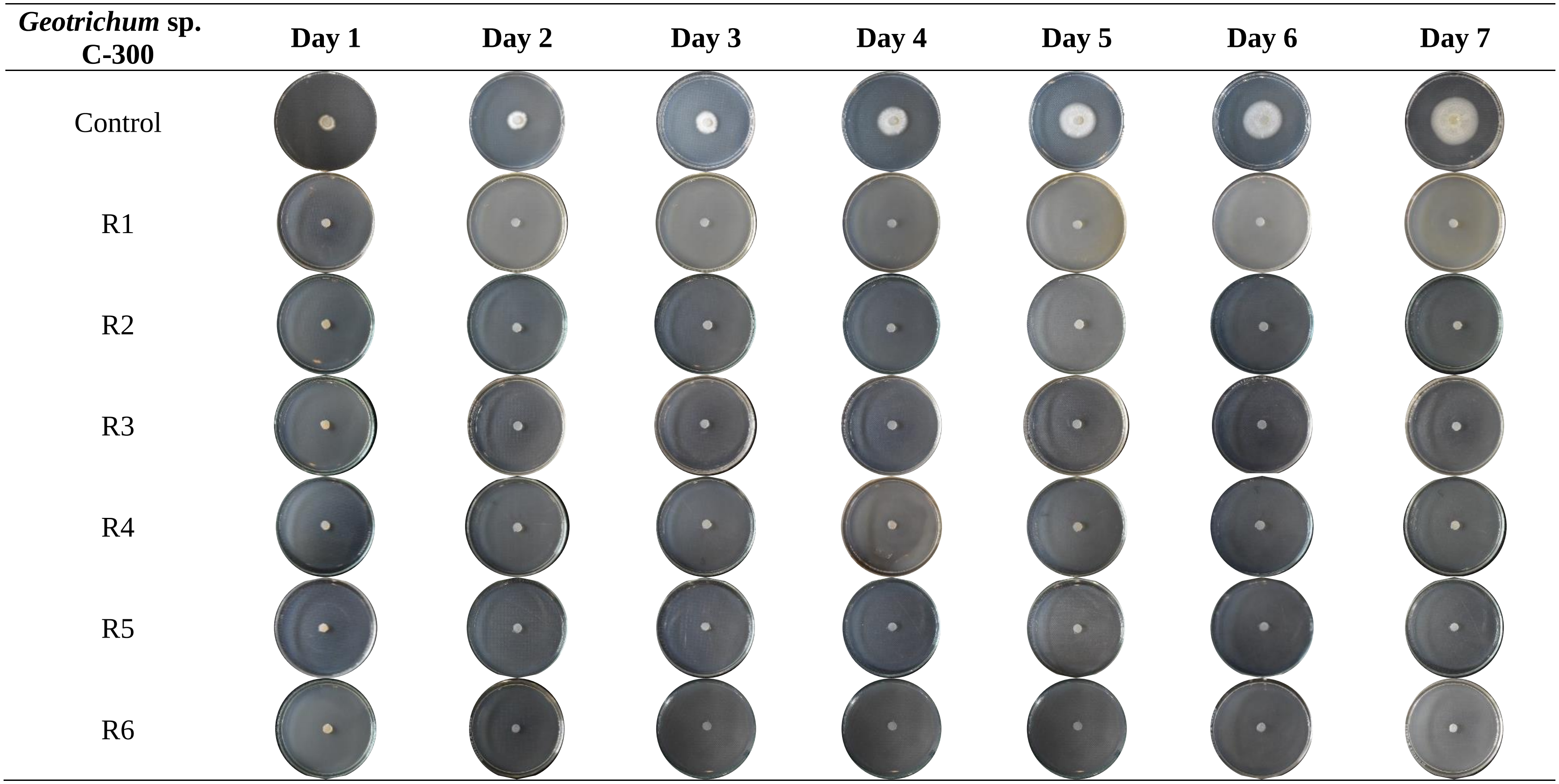

Figure S12. Effect on fungal growth of *Geotrichum* sp. after treatment with 300 ppm of cinnamon oil. R1-R6: repetitions

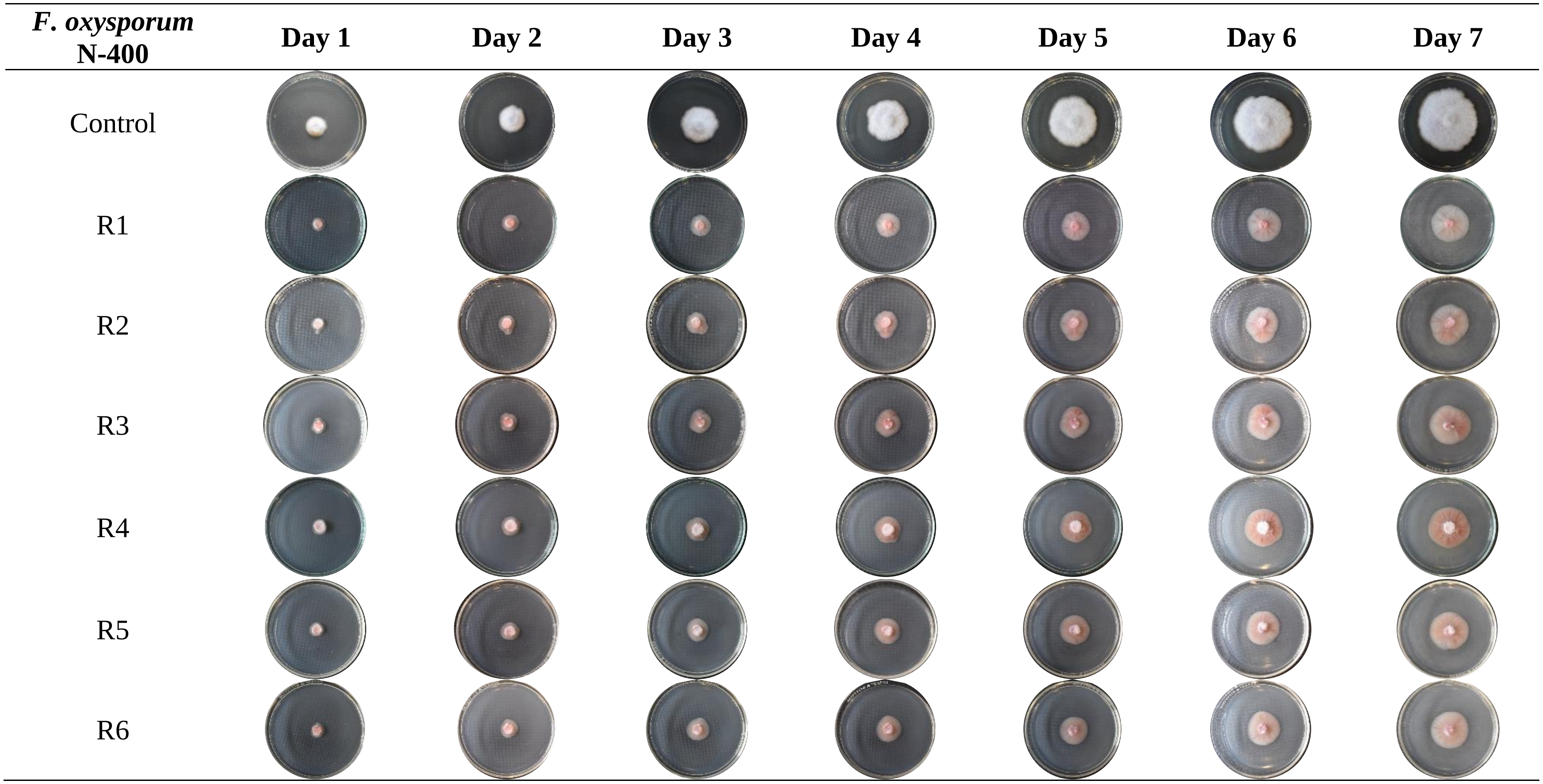

Figure S13. Effect on fungal growth of *Fusarium oxysporum* after treatment with 400 ppm of neem oil. R1-R6: repetitions

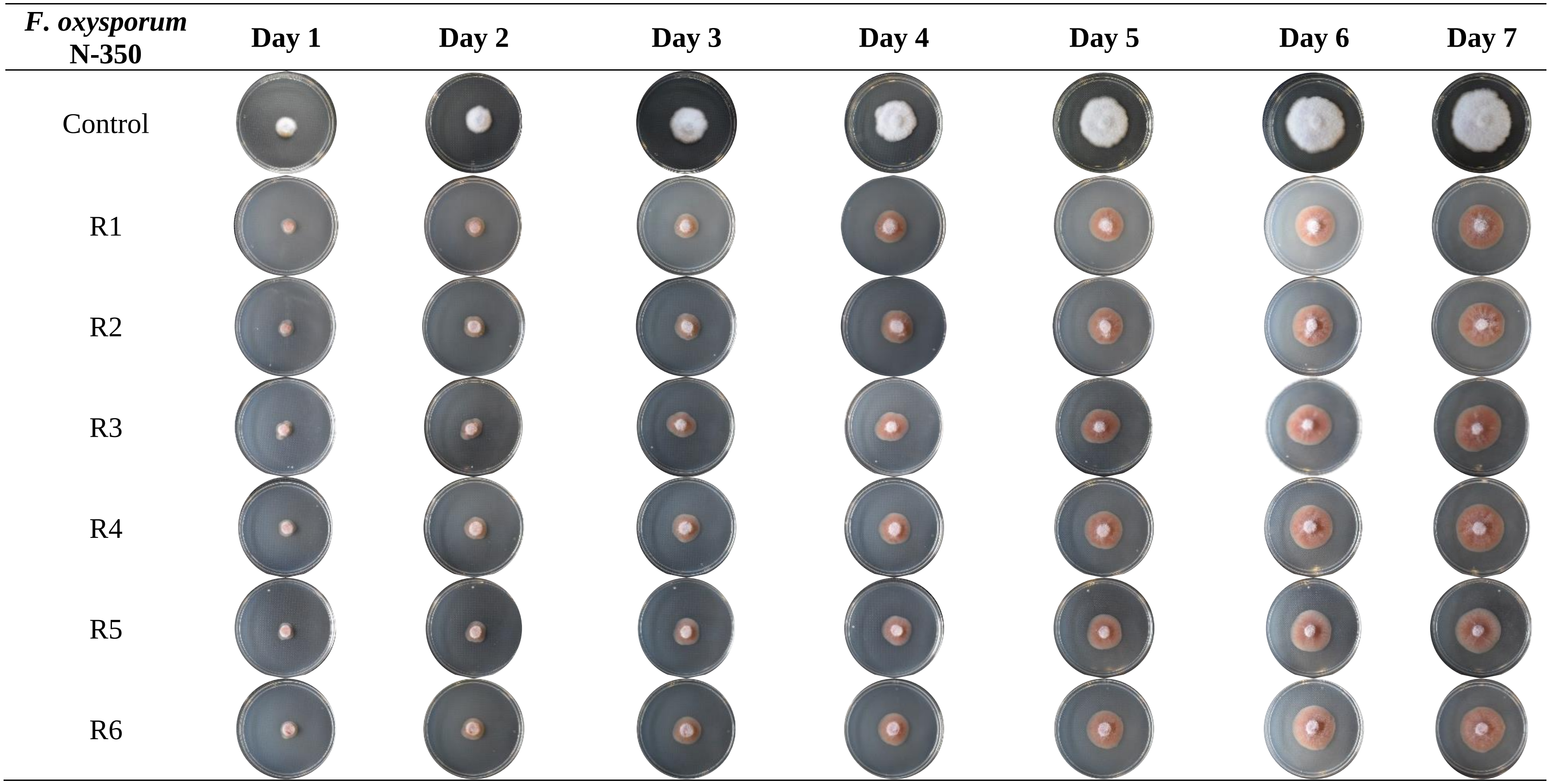

Figure S14. Effect on fungal growth of *Fusarium oxysporum* after treatment with 350 ppm of neem oil. R1-R6: repetitions

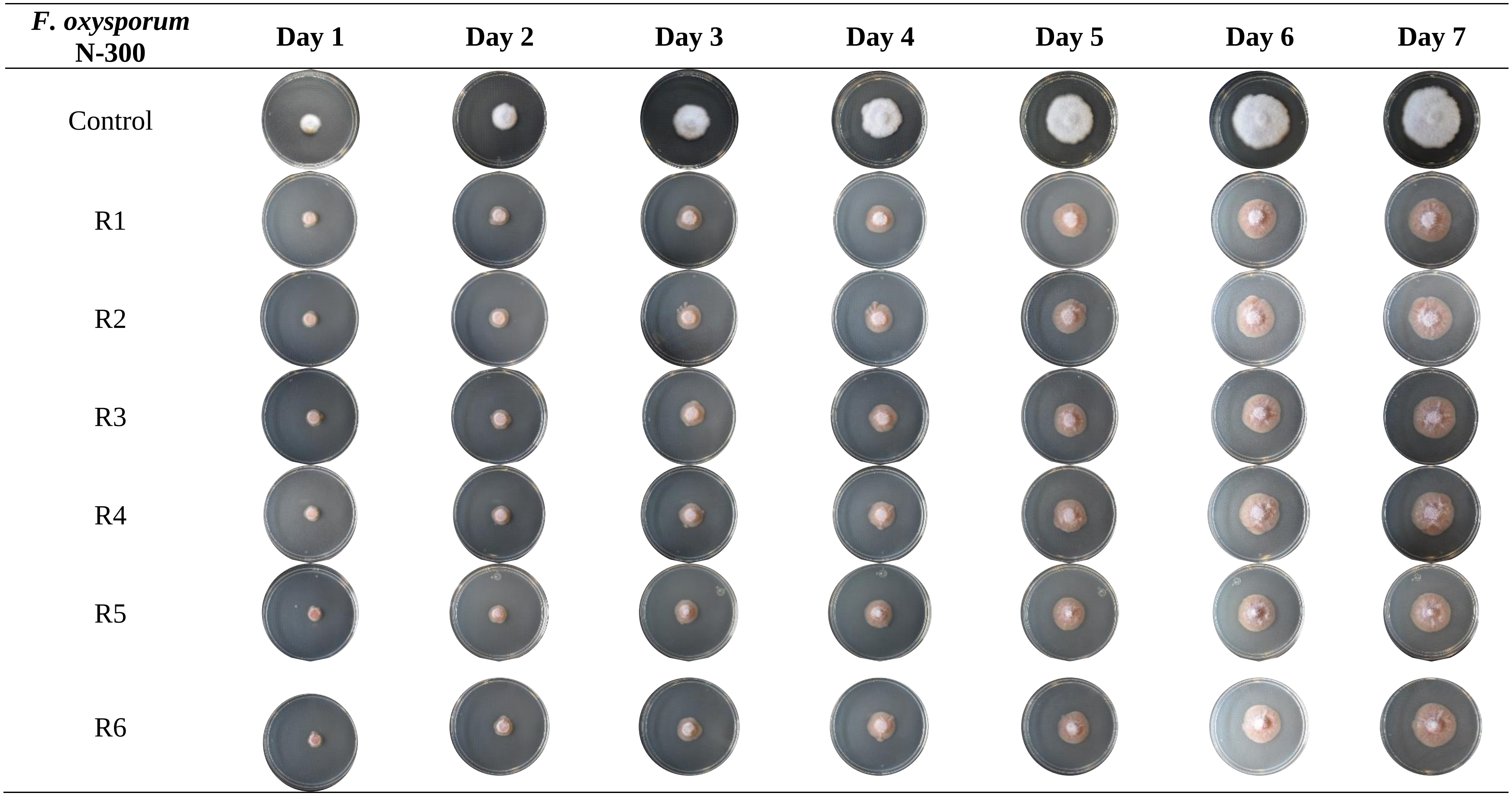

Figure S15. Effect on fungal growth of *Fusarium oxysporum* after treatment with 300 ppm of neem oil. R1-R6: repetitions

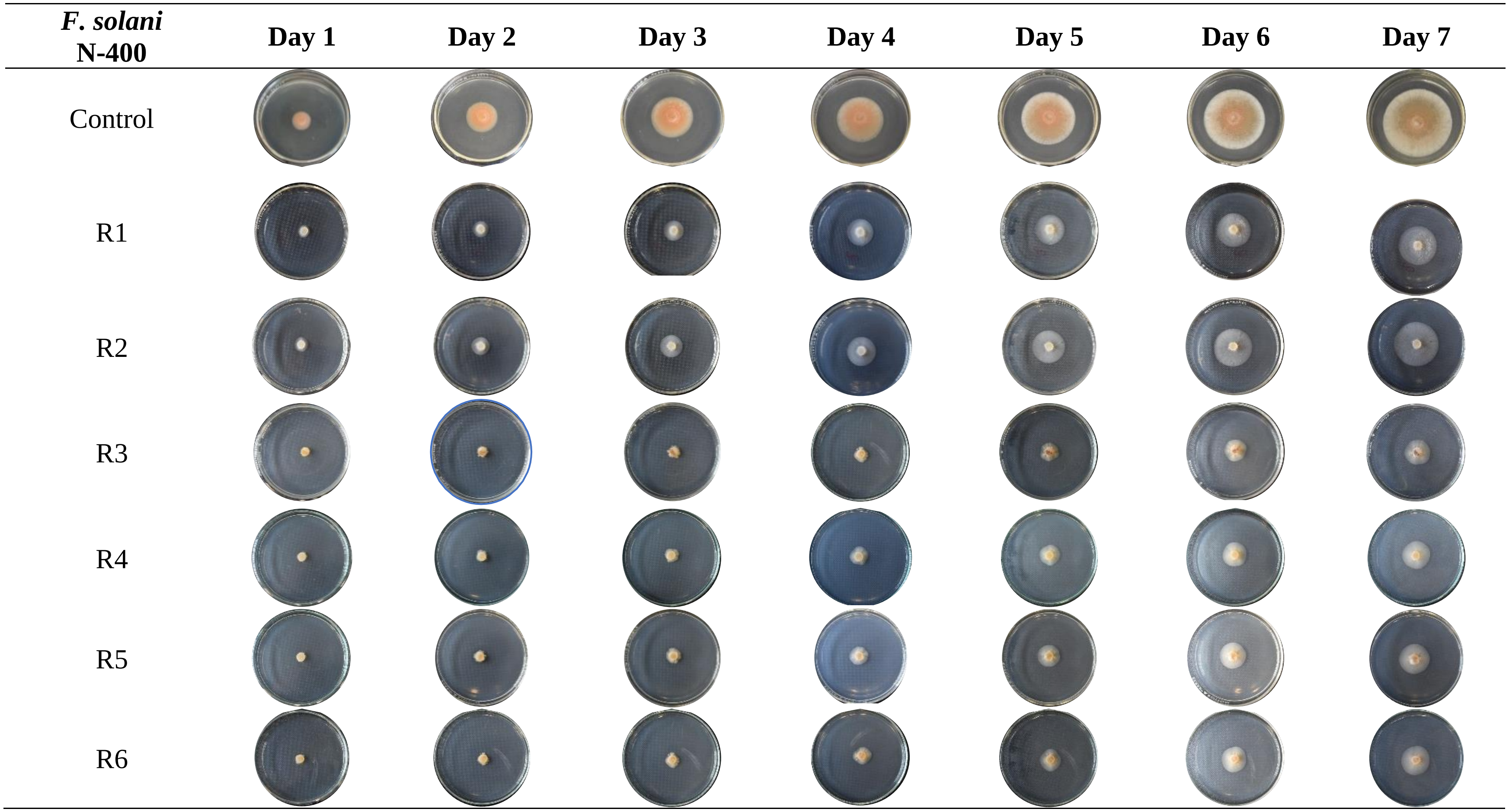

Figure S16. Effect on fungal growth of *Fusarium solani* after treatment with 400 ppm of neem oil. R1-R6: repetitions

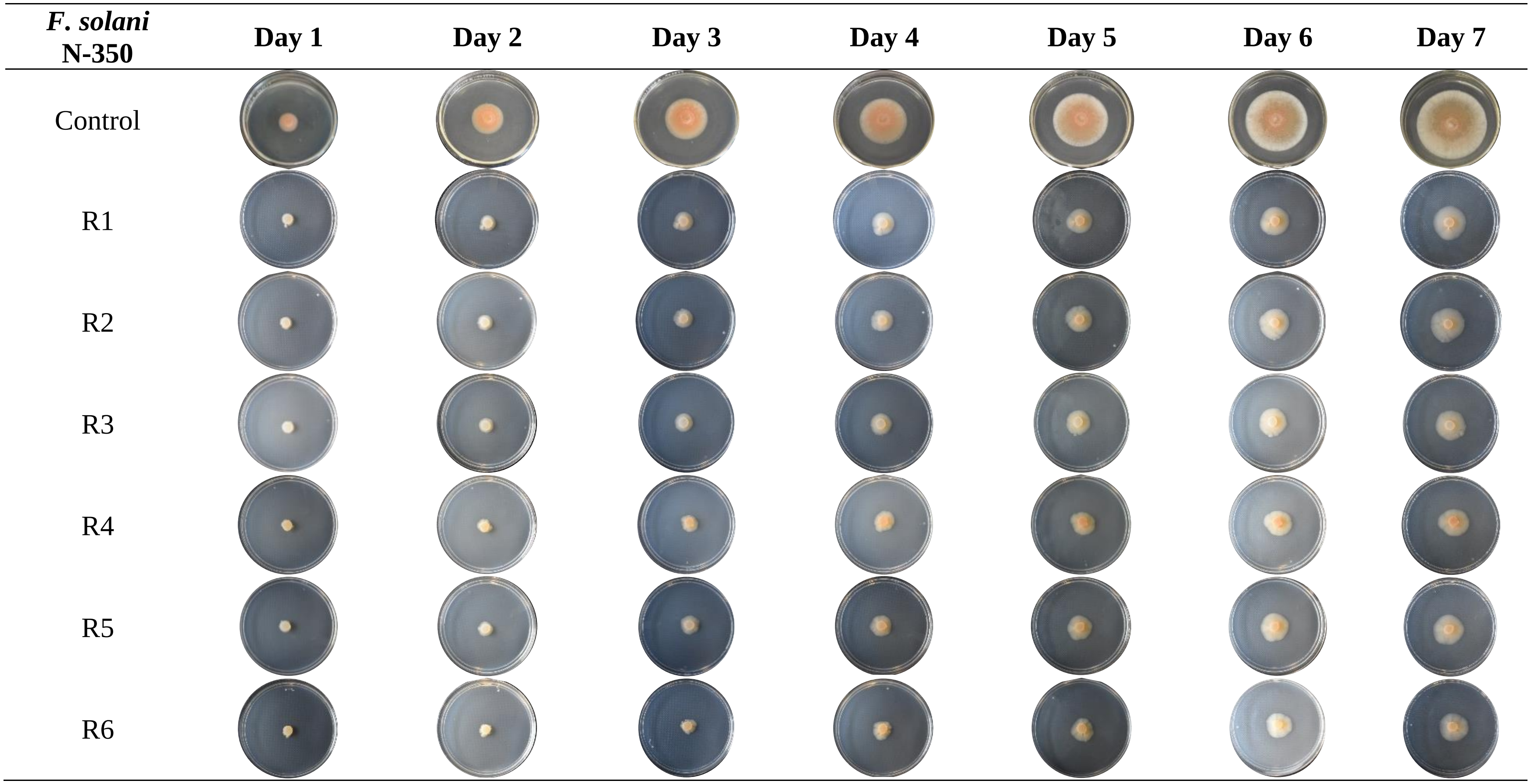

Figure S17. Effect on fungal growth of *Fusarium solani* after treatment with 350 ppm of neem oil. R1-R6: repetitions

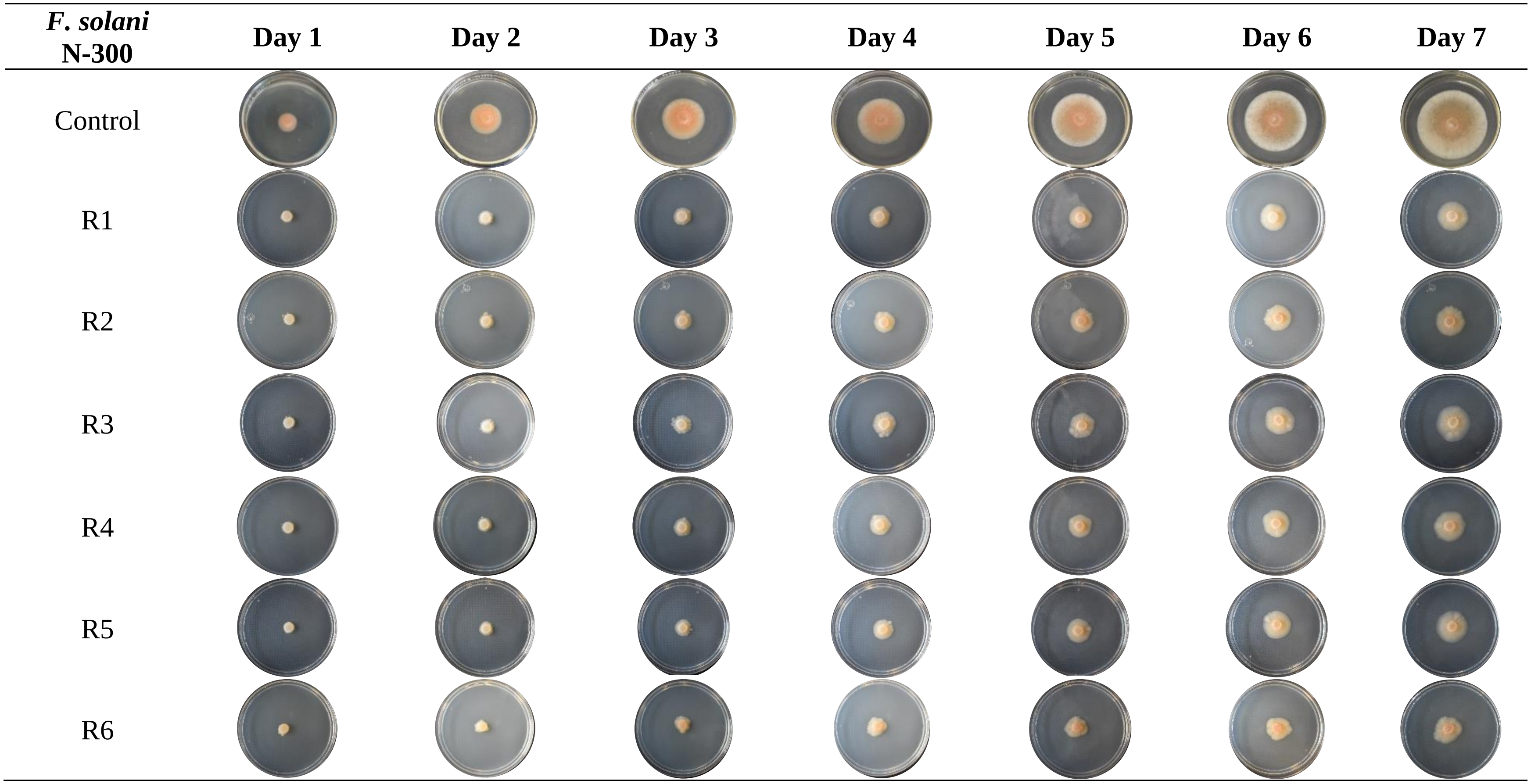

Figure S18. Effect on fungal growth of *Fusarium solani* after treatment with 300 ppm of neem oil. R1-R6: repetitions

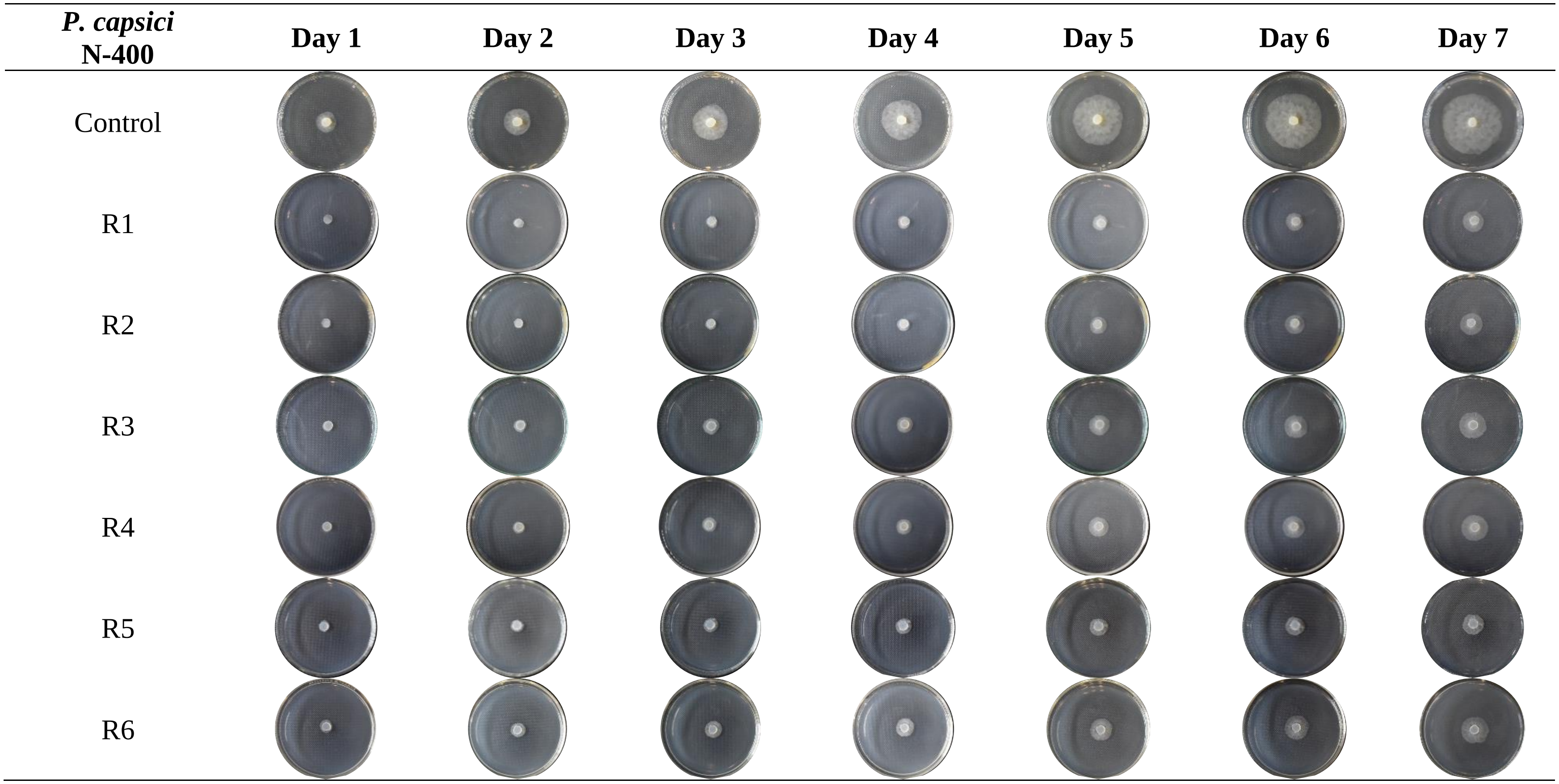

Figure S19. Effect on fungal growth of *Phytophthora capsici* after treatment with 400 ppm of neem oil. R1-R6: repetitions

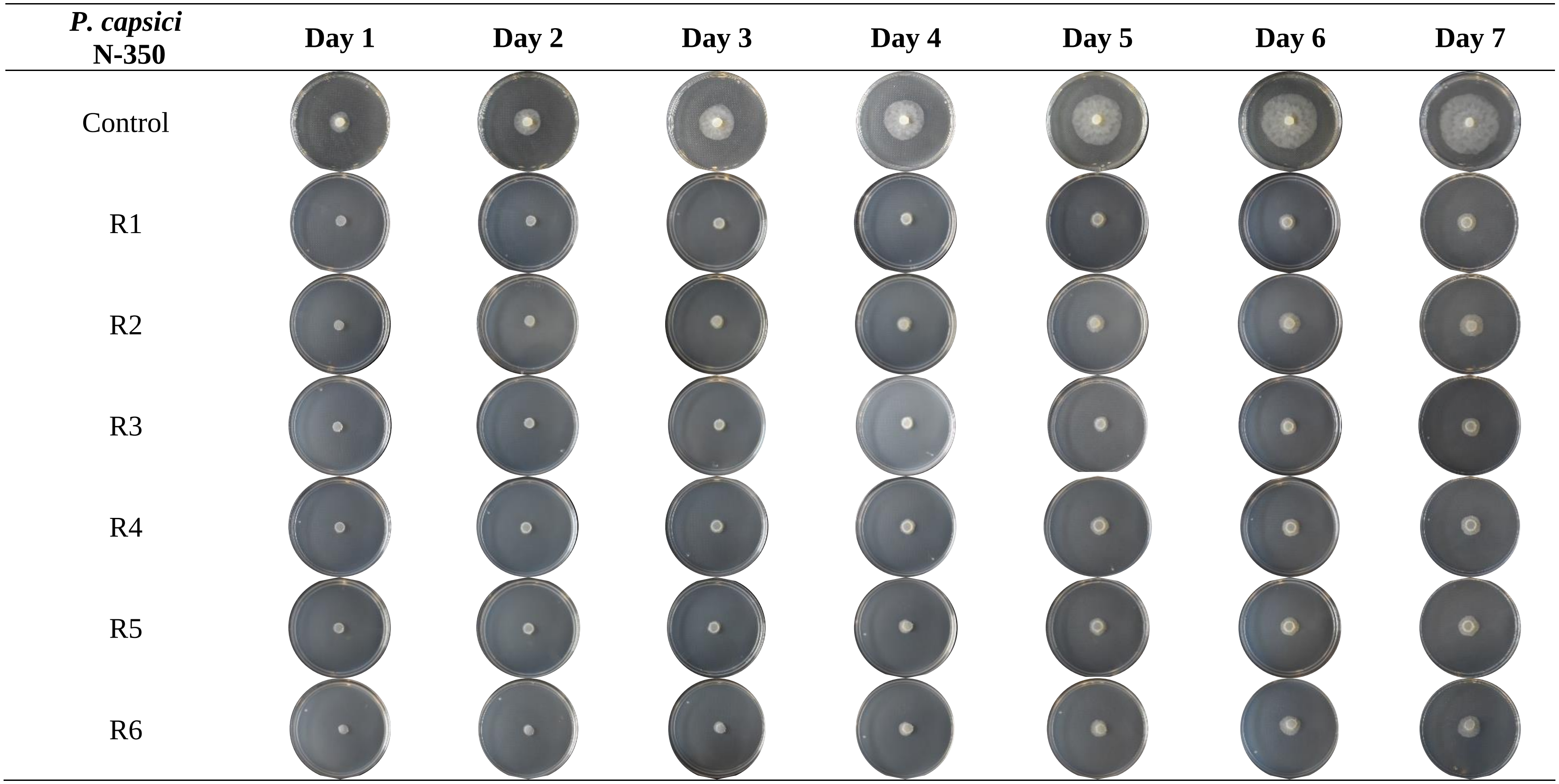

Figure S20. Effect on fungal growth of *Phytophthora capsici* after treatment with 350 ppm of neem oil. R1-R6: repetitions

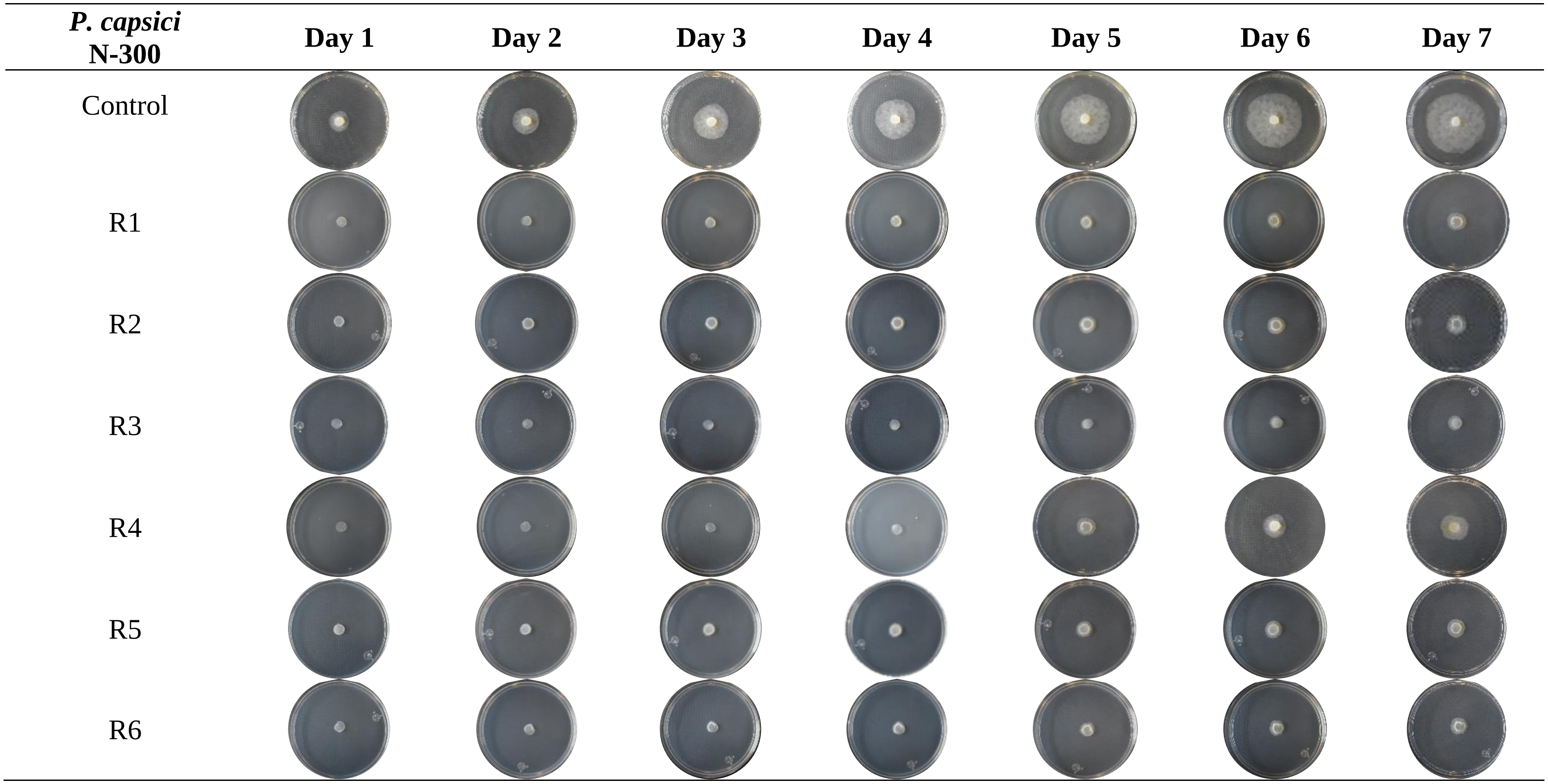

Figure S21. Effect on fungal growth of *Phytophthora capsici* after treatment with 300 ppm of neem oil. R1-R6: repetitions

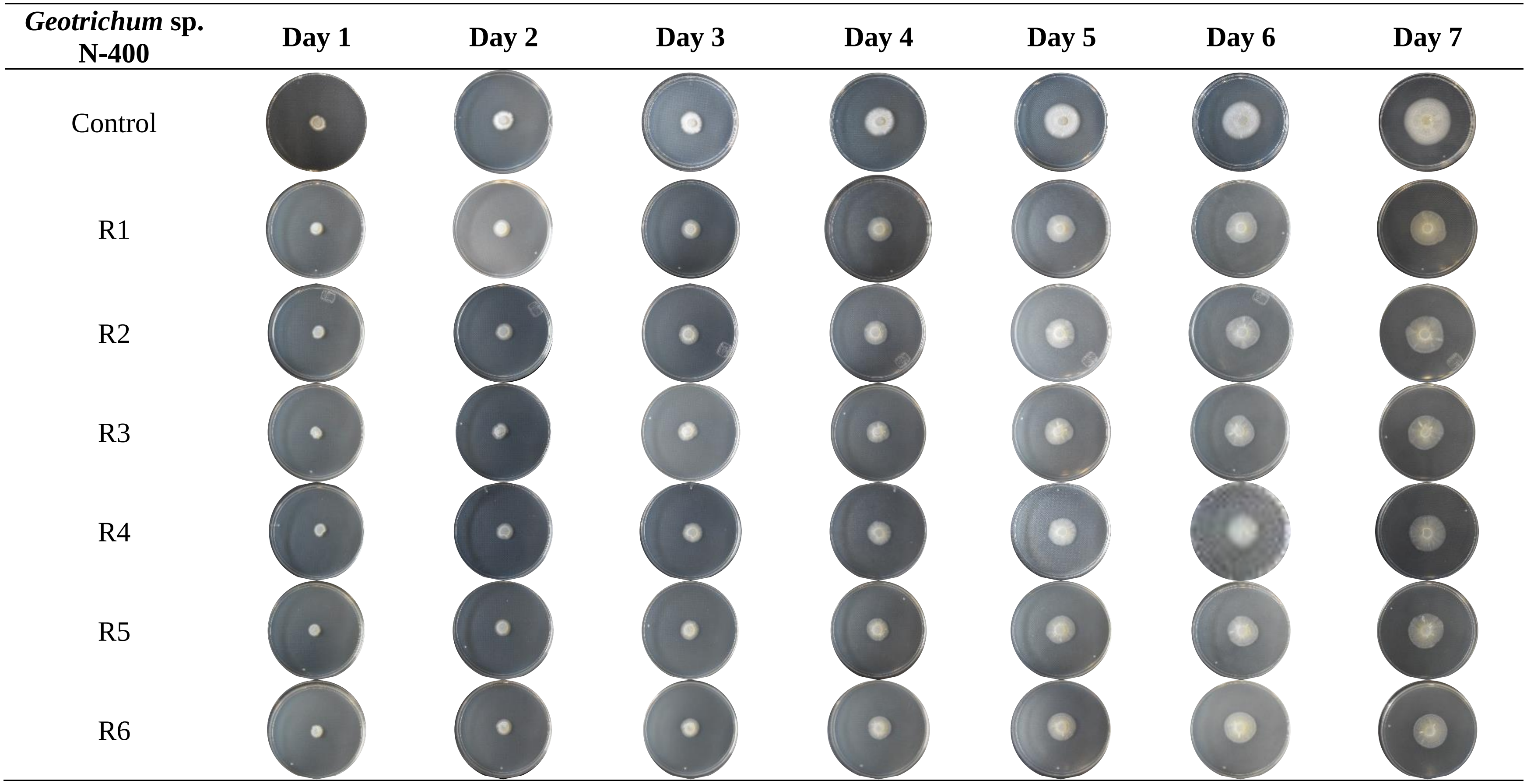

Figure S22. Effect on fungal growth of *Geotrichum* sp. after treatment with 400 ppm of neem oil. R1-R6: repetitions

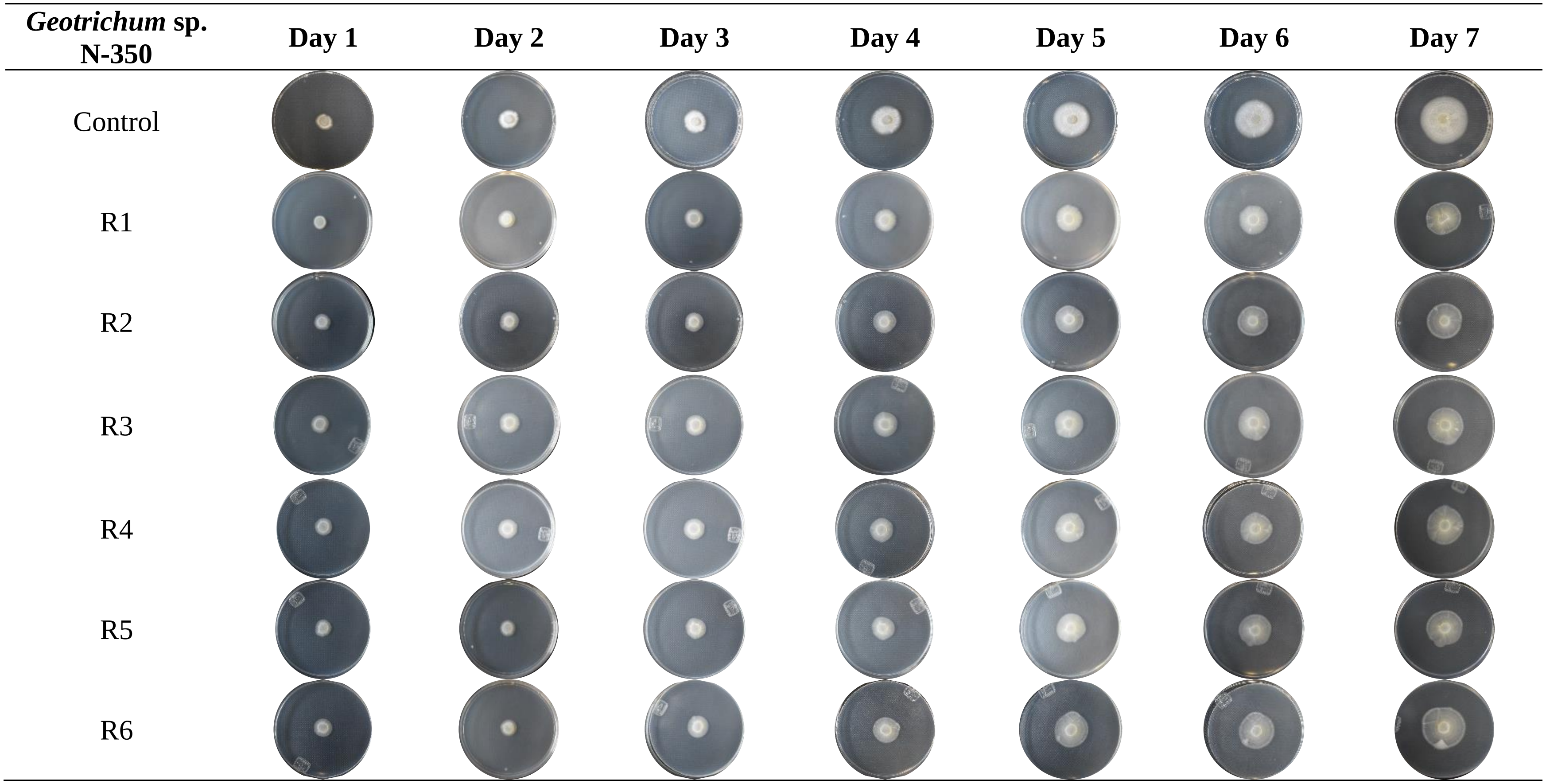

Figure S23. Effect on fungal growth of *Geotrichum* sp. after treatment with 350 ppm of neem oil. R1-R6: repetitions

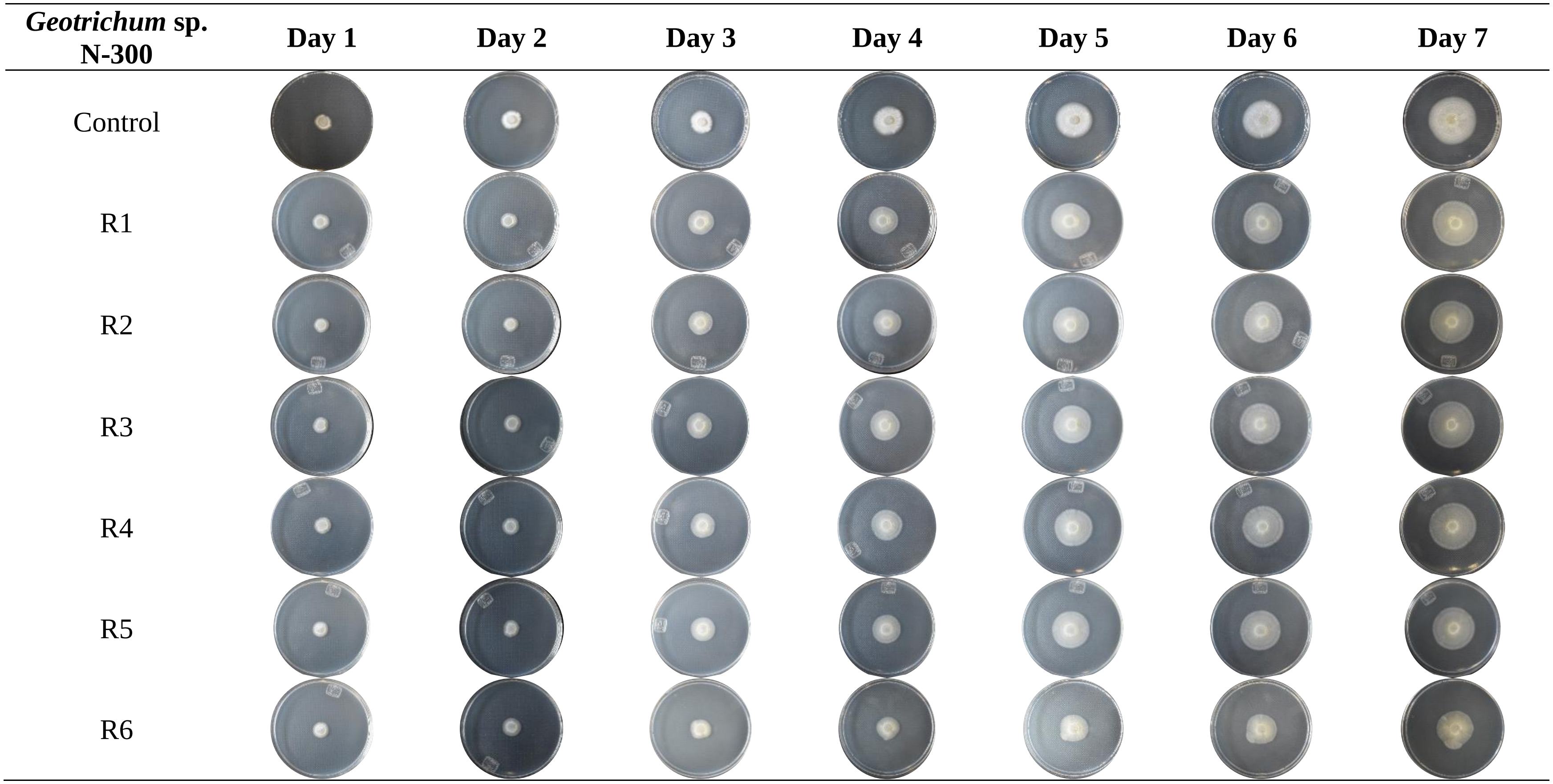

Figure S24. Effect on fungal growth of *Geotrichum* sp. after treatment with 300 ppm of neem oil. R1-R6: repetitions

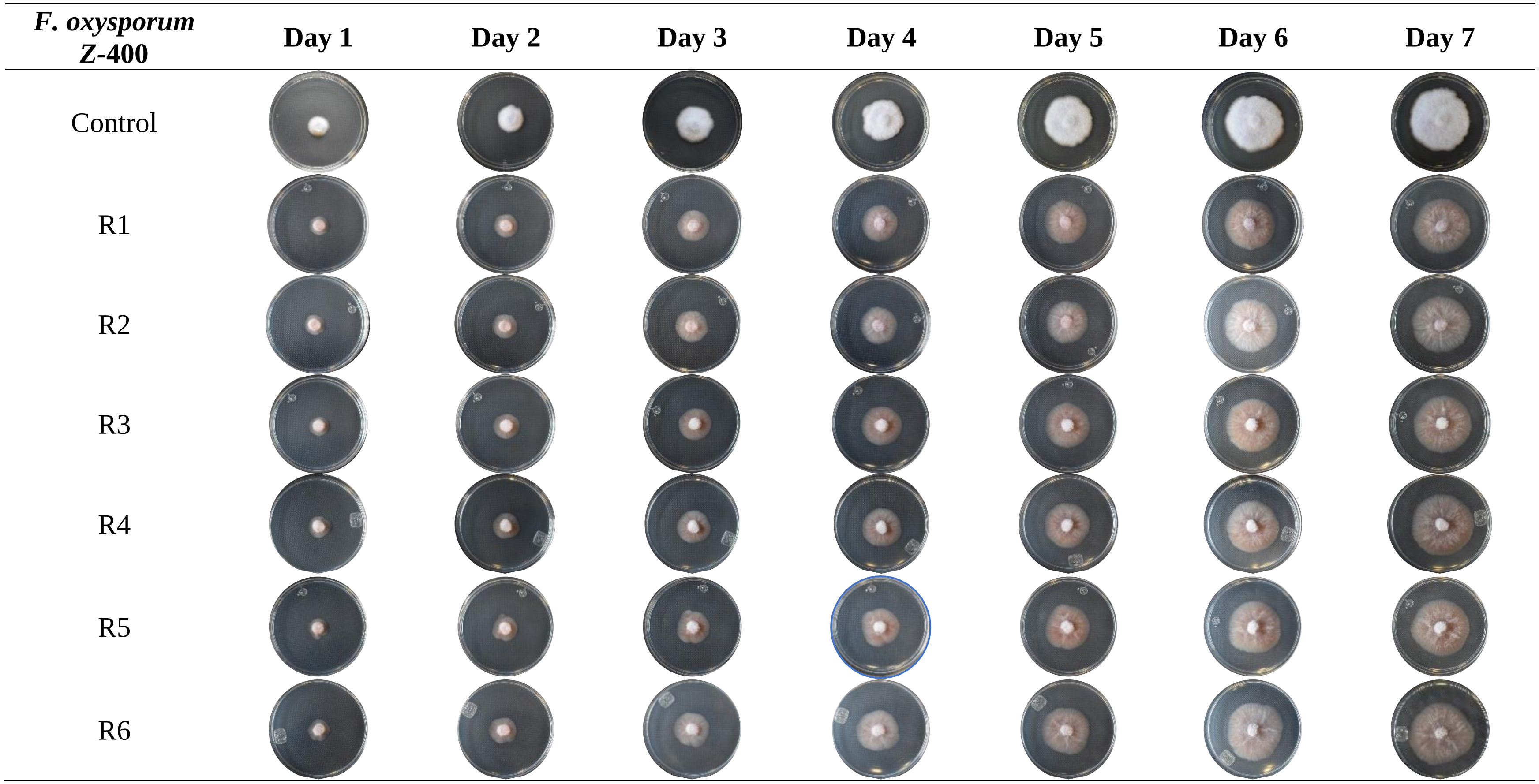

Figure S25. Effect on fungal growth of *Fusarium oxysporum* after treatment with 400 ppm of black sapote extract. R1-R6: repetitions

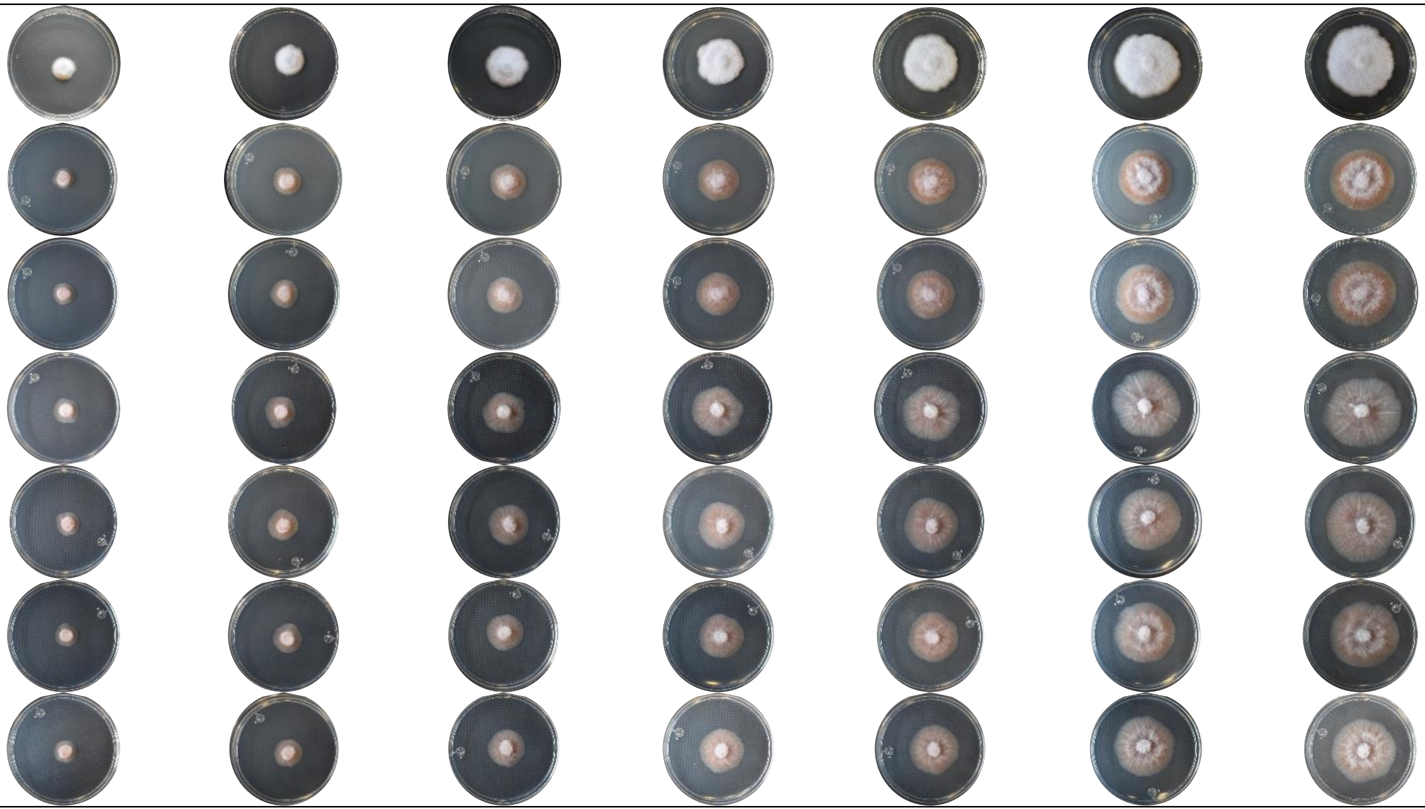

F. oxysporum Z-350	Day 1	Day 2	Day 3	Day 4	Day 5	Day 6	Day 7
Control							
R1							
R2							
R3							
R4							
R5							
R6							

Figure S26. Effect on fungal growth of *Fusarium oxysporum* after treatment with 350 ppm of black sapote extract. R1-R6: repetitions

F. oxysporum Z-300	Day 1	Day 2	Day 3	Day 4	Day 5	Day 6	Day 7
Control	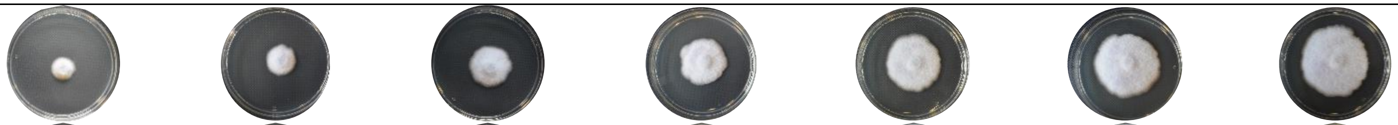						
R1	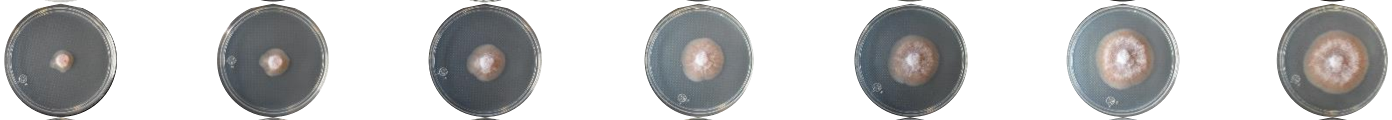						
R2	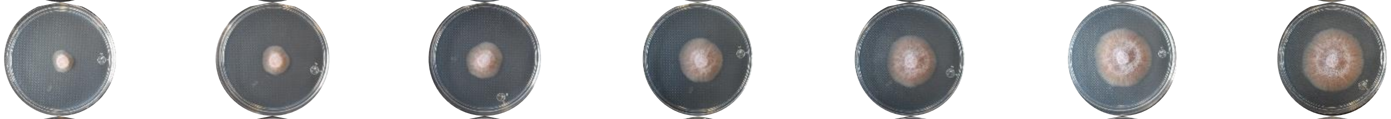						
R3	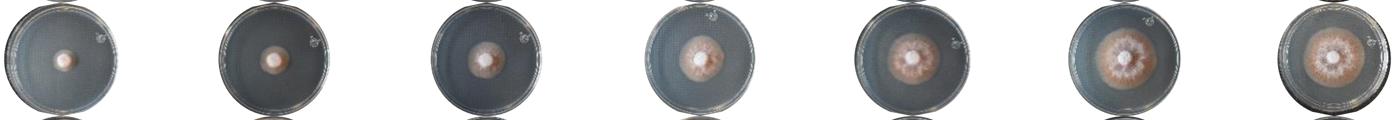						
R4	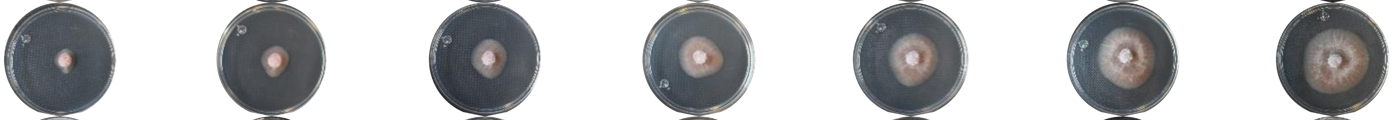						
R5	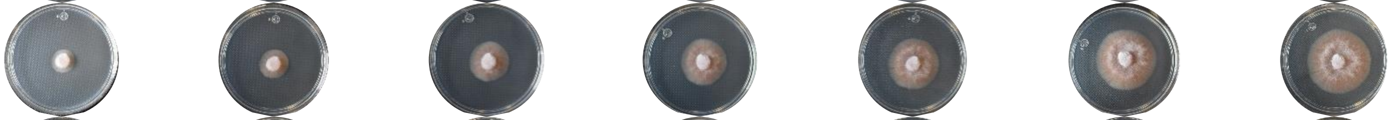						
R6	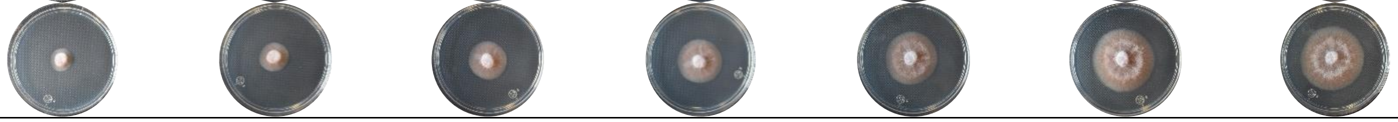						

Figure S27. Effect on fungal growth of *Fusarium oxysporum* after treatment with 300 ppm of black sapote extract. R1-R6: repetitions

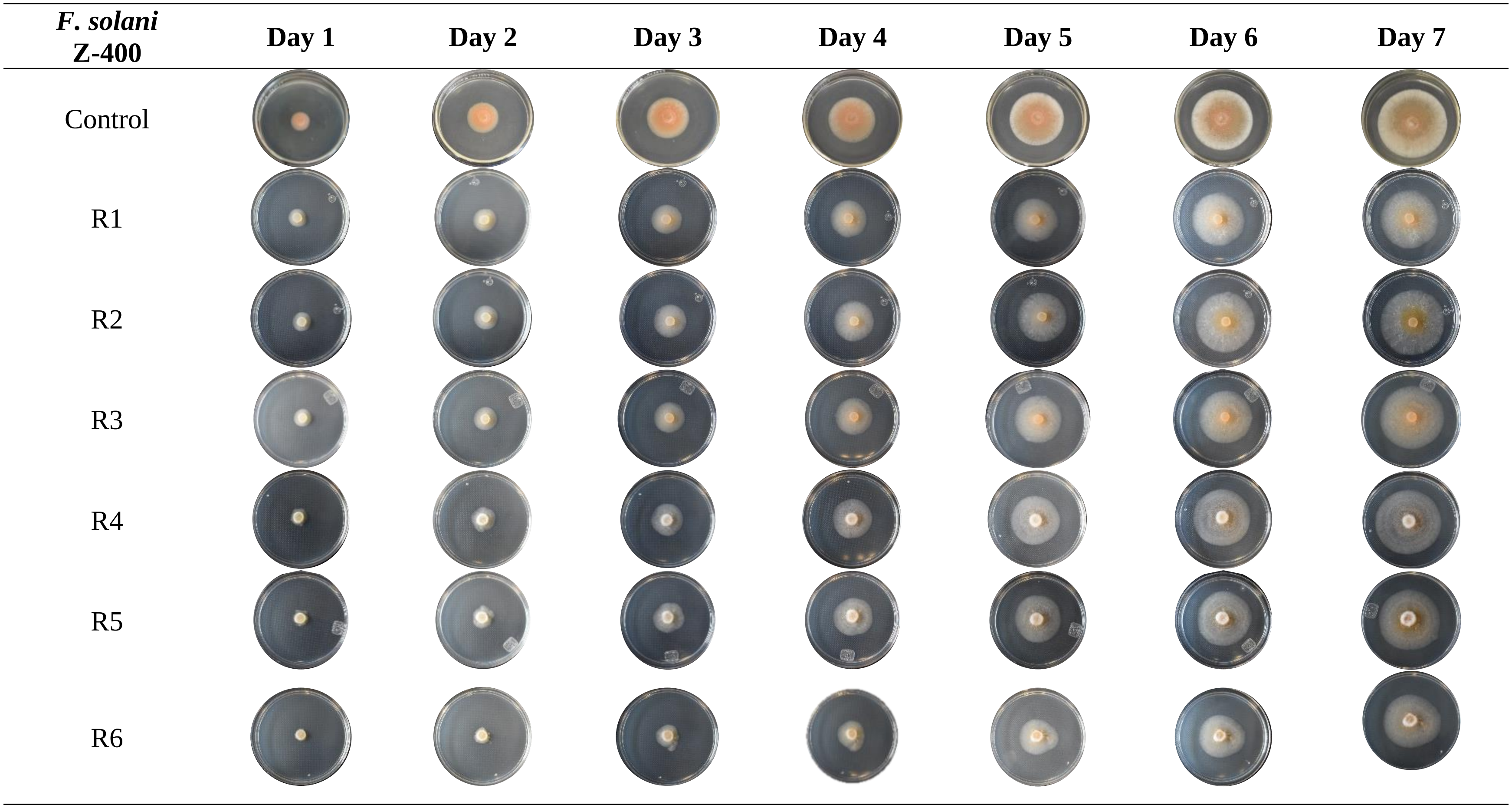

Figure S28. Effect on fungal growth of *Fusarium solani* after treatment with 400 ppm of black sapote extract. R1-R6: repetitions

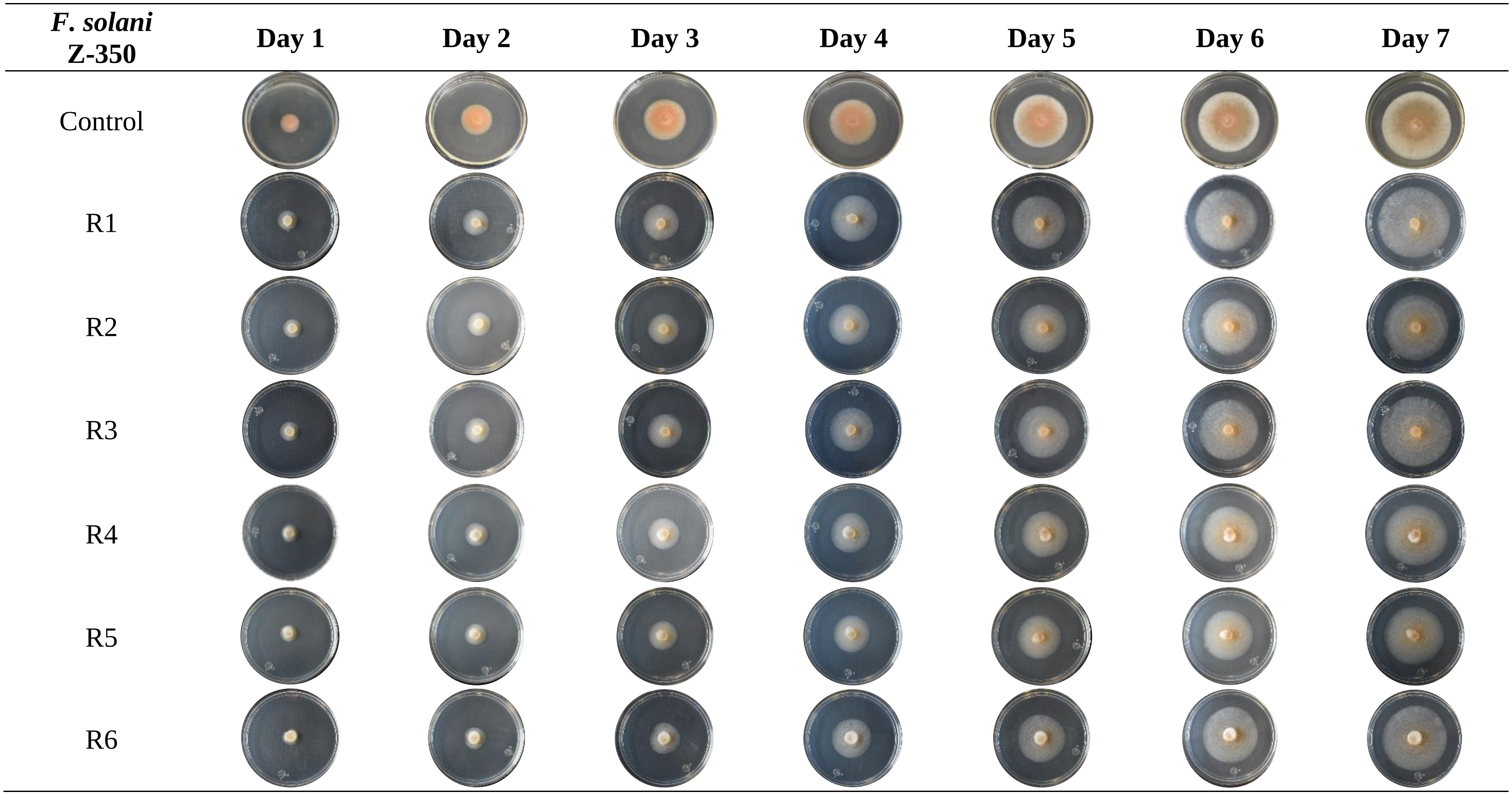

Figure S29. Effect on fungal growth of *Fusarium solani* after treatment with 350 ppm of black sapote extract. R1-R6: repetitions

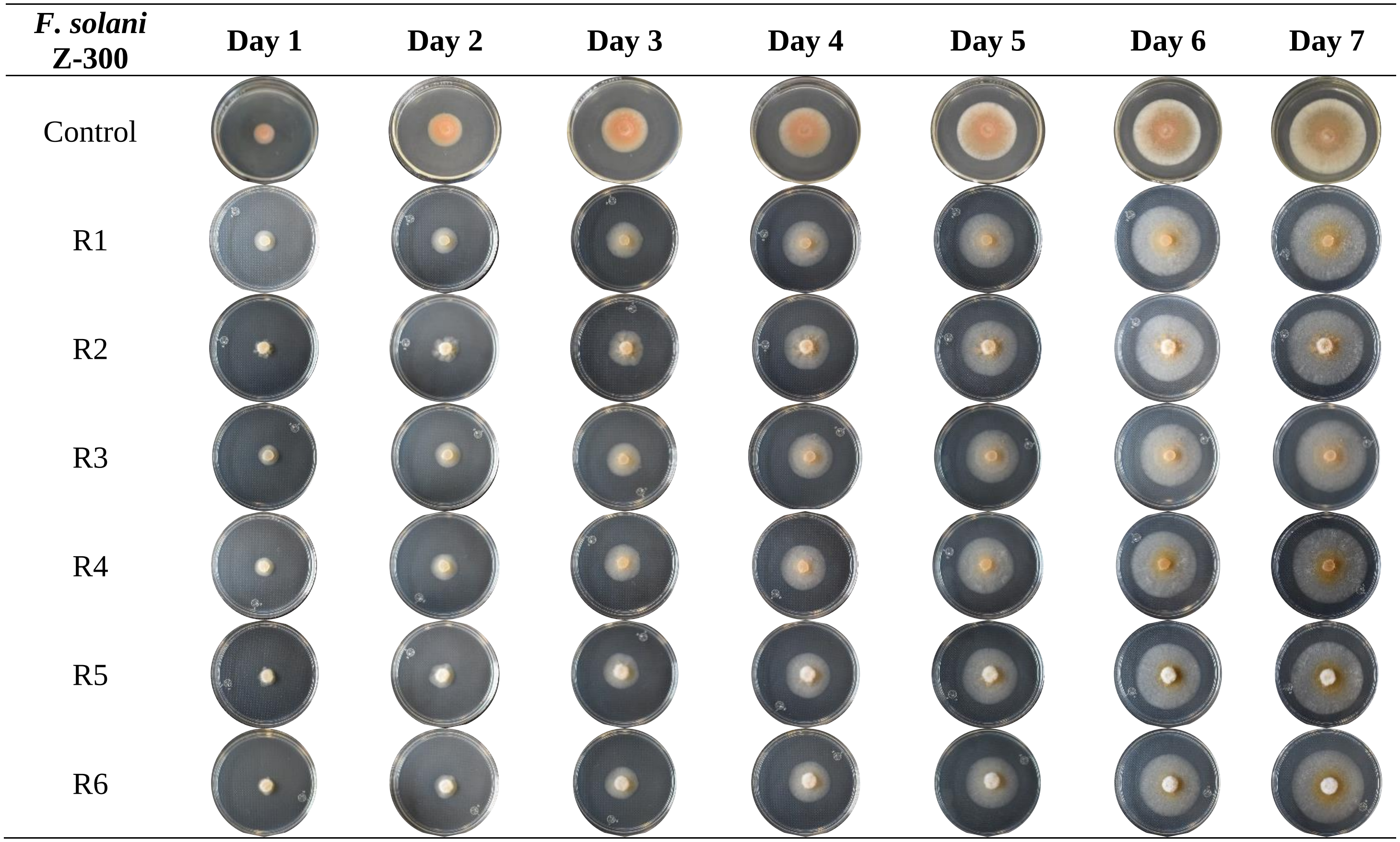

Figure S30. Effect on fungal growth of *Fusarium solani* after treatment with 300 ppm of black sapote extract. R1-R6: repetitions

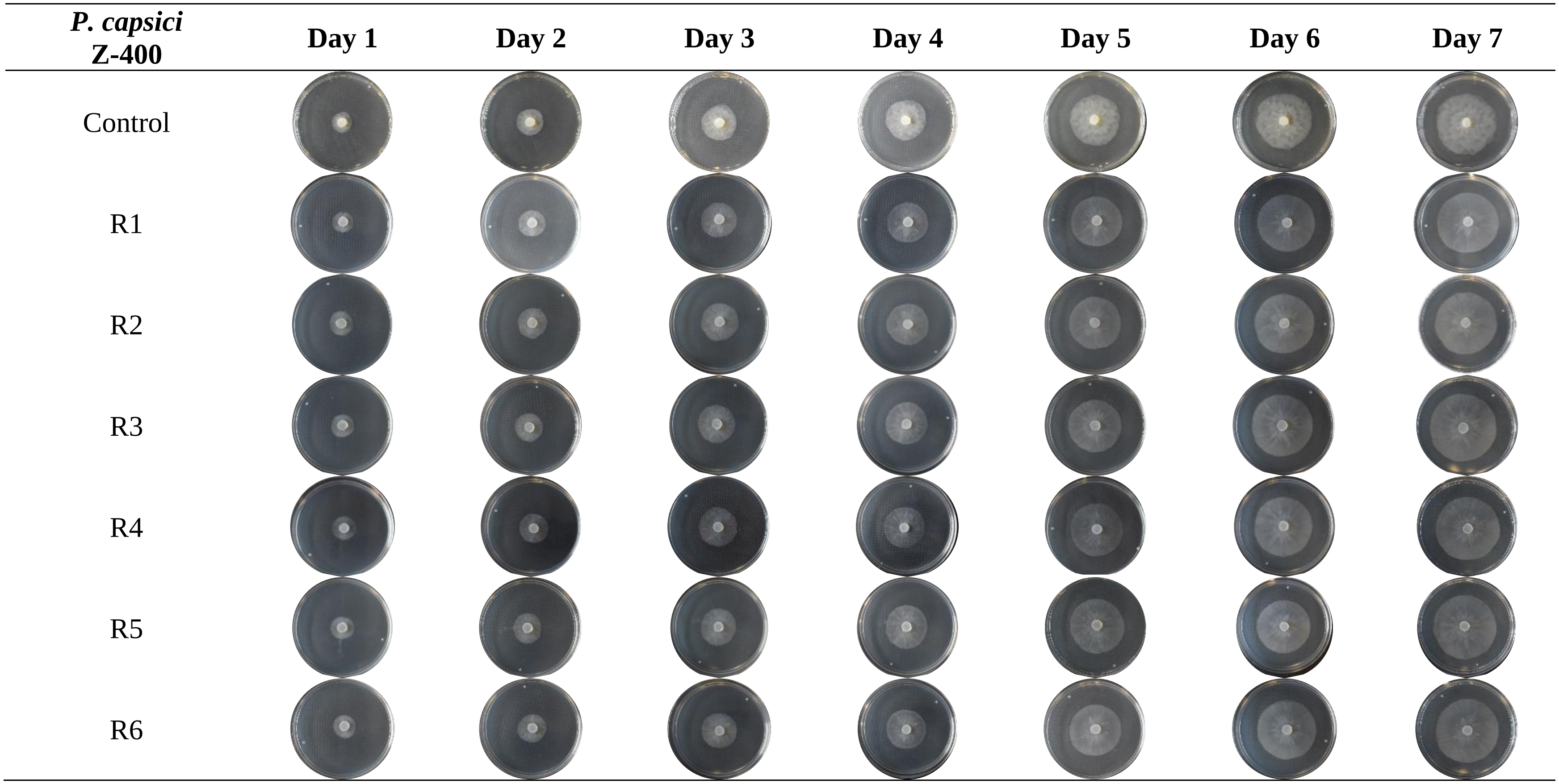

Figure S31. Effect on fungal growth of *Phytophthora capsici* after treatment with 400 ppm of black sapote extract. R1-R6: repetitions

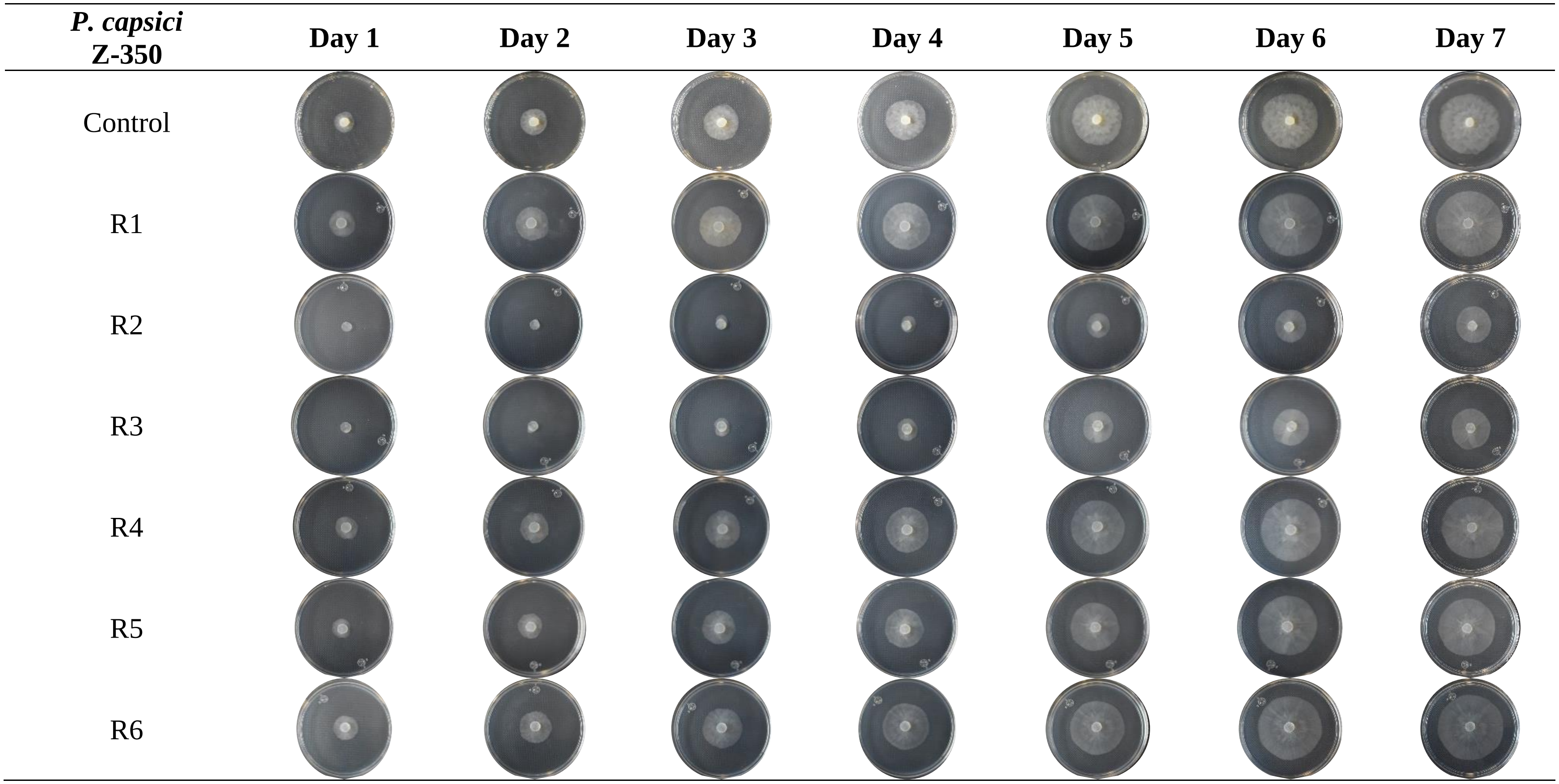

Figure S32. Effect on fungal growth of *Phytophthora capsici* after treatment with 350 ppm of black sapote extract. R1-R6: repetitions

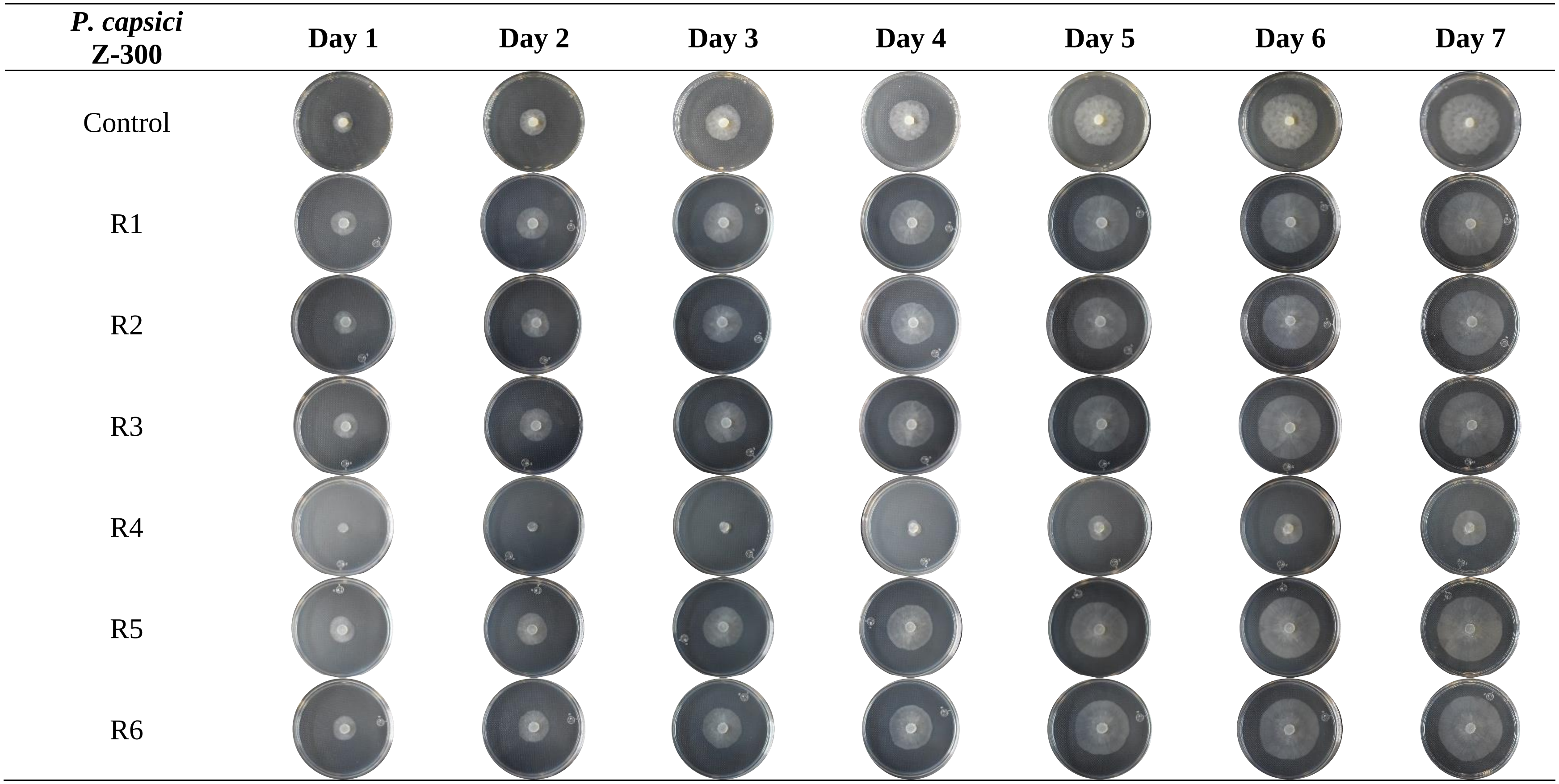

Figure S33. Effect on fungal growth of *Phytophthora capsici* after treatment with 300 ppm of black sapote extract. R1-R6: repetitions

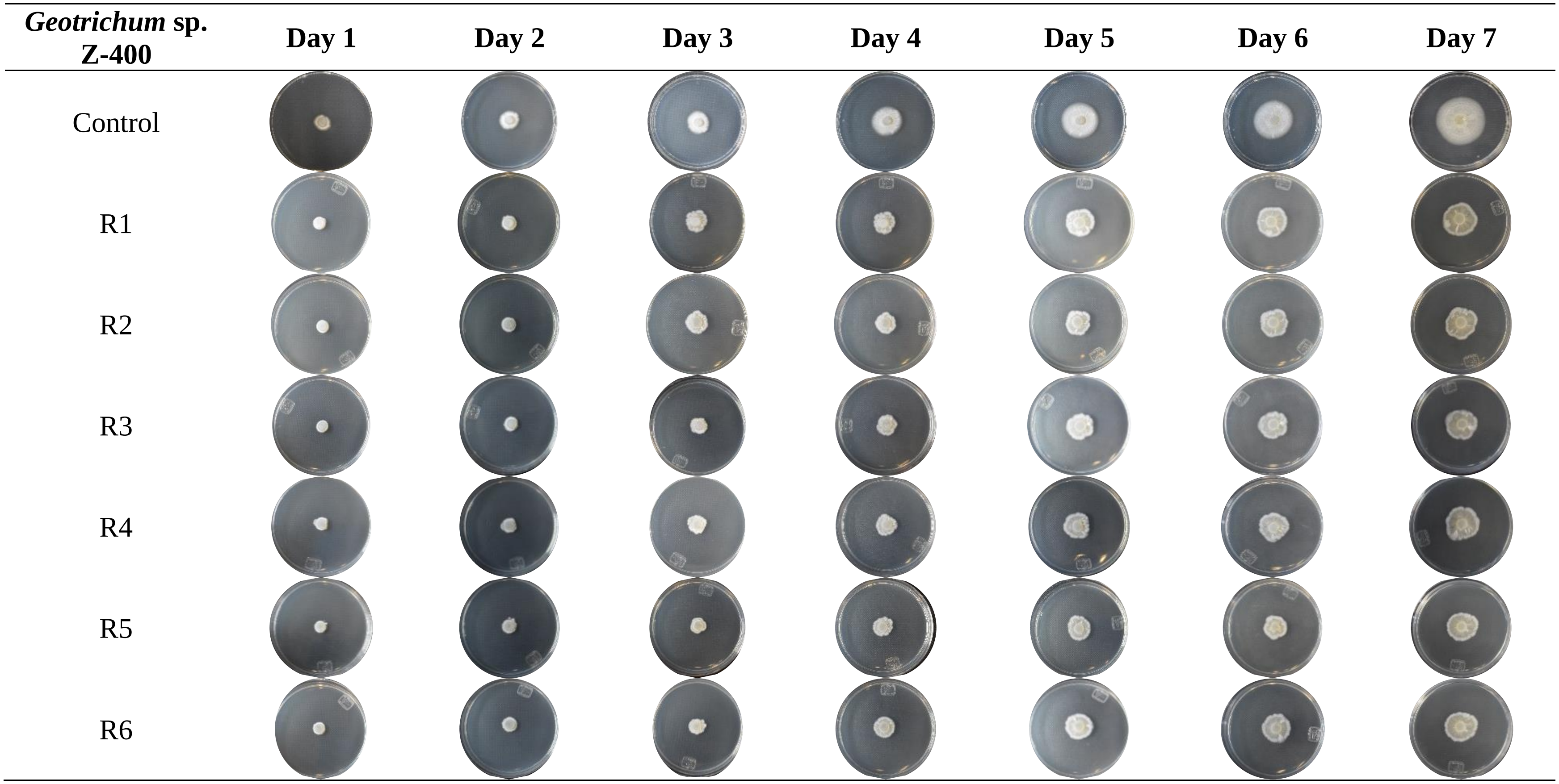

Figure S34. Effect on fungal growth of *Geotrichum* sp. after treatment with 400 ppm of black sapote extract. R1-R6: repetitions

Geotrichum sp. Z-350	Day 1	Day 2	Day 3	Day 4	Day 5	Day 6	Day 7
Control							
R1							
R2							
R3							
R4							
R5							
R6							

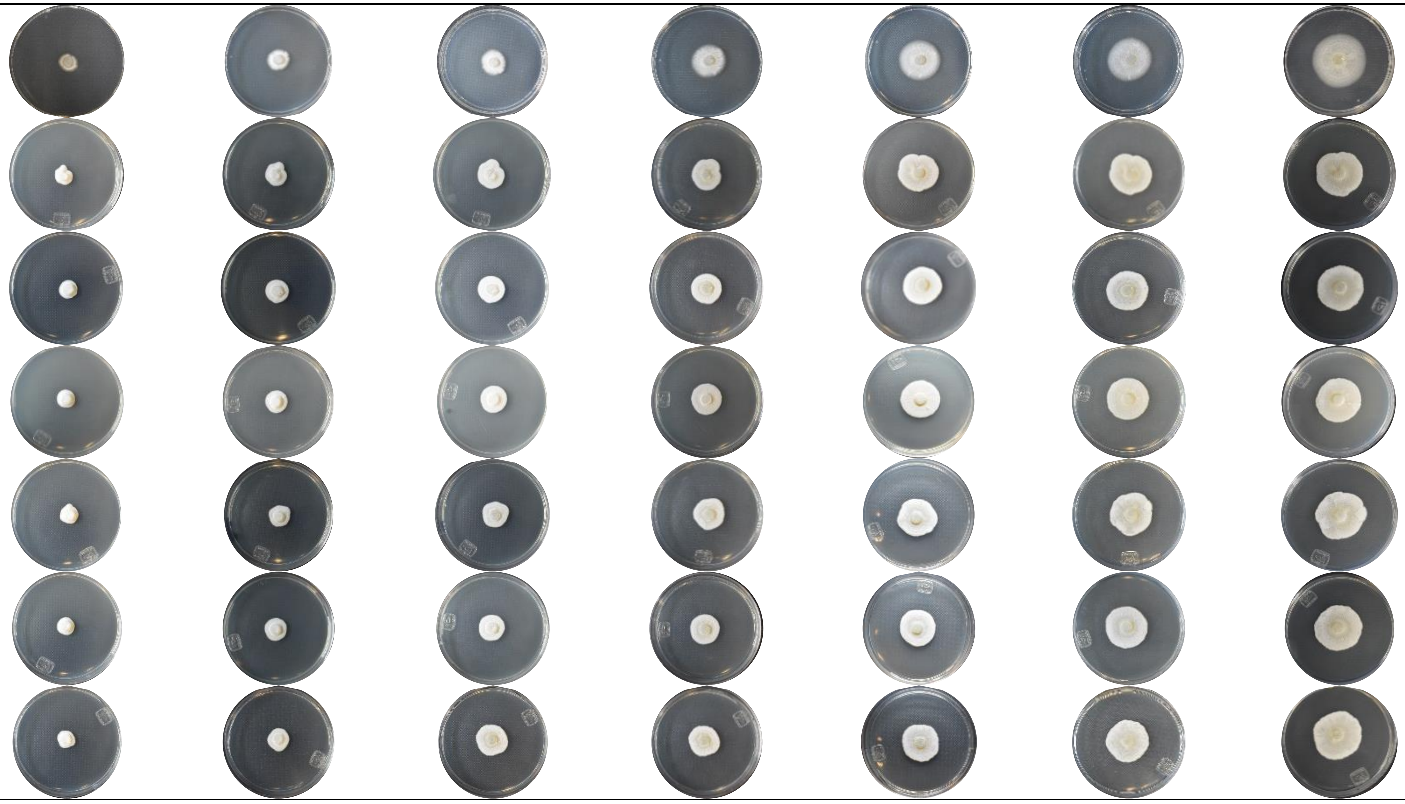

Figure S35. Effect on fungal growth of *Geotrichum* sp. after treatment with 350 ppm of black sapote extract. R1-R6: repetitions

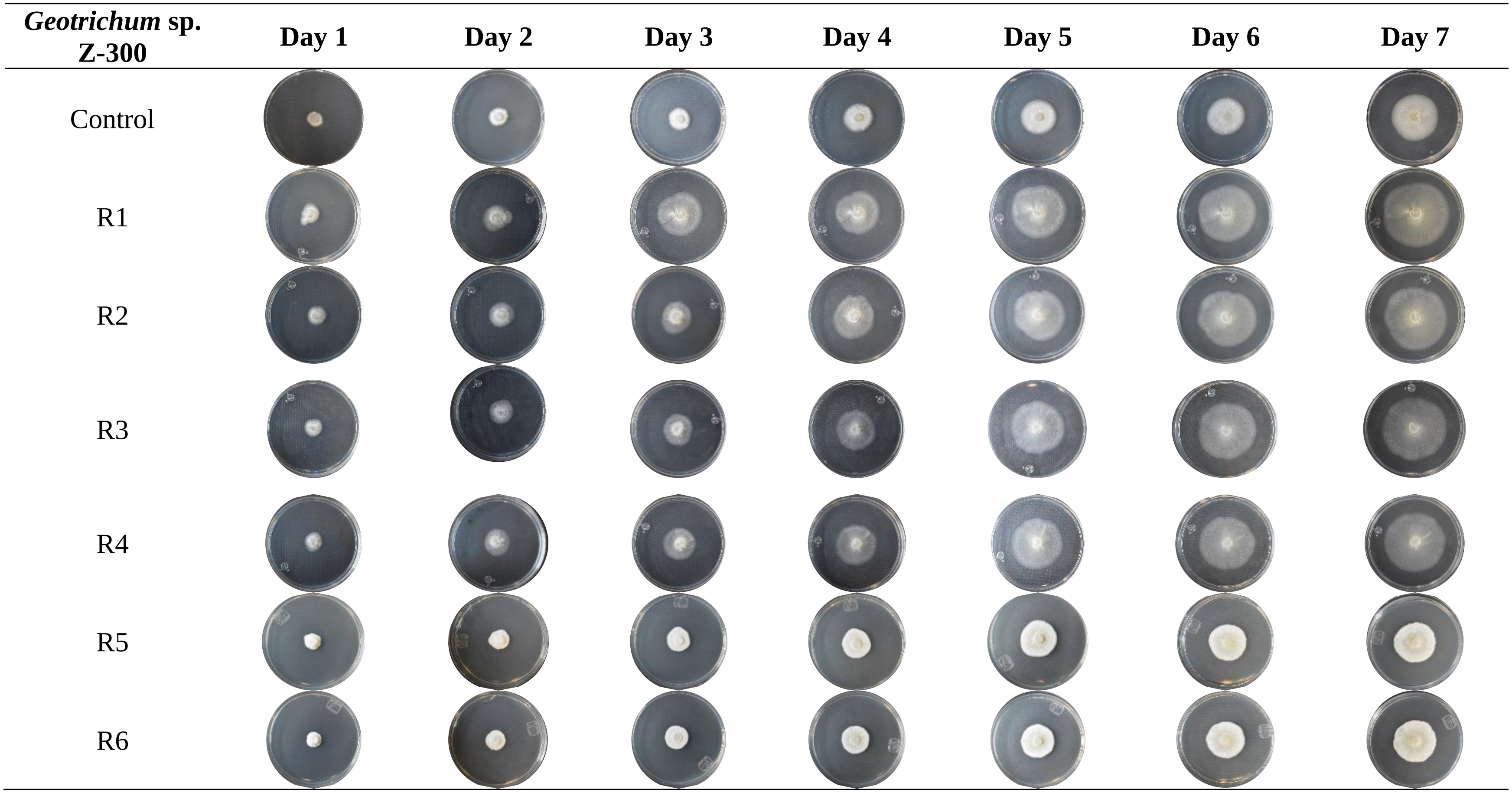

Figure S36. Effect on fungal growth of *Geotrichum* sp. after treatment with 300 ppm of black sapote extract. R1-R6: repetitions
